# Supplementary material for: A method to construct the dynamic landscape of a bio-membrane with experiment and simulation
Source: Nat Commun. 2022 Jan 10;13:108. doi: 10.1038/s41467-021-27417-y (PMC8748619; doi:10.1038/s41467-021-27417-y)
Supplement: Supplementary file 1 — Supplementary Information [file 41467_2021_27417_MOESM1_ESM.pdf]

Supplementary information for:

A method to construct the dynamic landscape of a bio-membrane with experiment and simulation

Albert A. Smith[1*], Alexander Vogel[1], Oskar Engberg[1], Peter W. Hildebrand[1], Daniel Huster[1]

[1]*Institute for Medical Physics and Biophysics, Leipzig University, Härtelstr. 16-18, 04107 Leipzig, Germany*

*E-mail: albert.smith-penzel@medizin.uni-leipzig.de

Supplementary Note 1: Experimental acquisition and data processing **2**
- *1.1 Assignment and initial spectrum fits* 2
- *1.2 Experiments* 2
- *1.3 Extraction of rate constants* 4
- *1.4 Detector analysis* 6
- *1.5 Experimental and Simulated Detector Optimization* 9
- *1.6 Experimental data fits* 10
- *1.7 Additional Information on Detector Analysis* 14
- *1.8 Comparison of MD simulations* 15

Supplementary Note 2: Frame analysis **16**
- *2.1 Definition of the frames of NMR interactions* 19
- *2.2 Definition of RMS frames* 19
- *2.3 Definition of MOI frames* 20
- *2.4 Definition of bond frames* 20
- *2.5 Separating parallel and perpendicular motion: MOIxy frame* 20
- *2.6 Frame screening* 21

Supplementary Note 3: Movie descriptions **23**
- *3.1 Time indicator* 23
- *3.2 Plotting detector responses* 23
- *3.3 Plotting tensors* 24

Supplementary Note 4: Dynamic Landscape construction **25**
- *4.1 Choice of functional form of the distribution* 25
- *4.2 Comparison of detector responses* 28

Supplementary Note 5: Further analysis **35**
- *5.1 Configurational dynamics of the glycerol backbone* 35
- 5.2 S^2 vs. $1/T_1$ via detectors 36

Supplementary References **37**

Supplementary Note 1: Experimental acquisition and data processing

1.1 Assignment and initial spectrum fits

POPC has been assigned previously,[1–3] with the ^{13}C assignment shown in Supplementary Figure 1. In order to extract experimental relaxation rate constants as accurately as possible, we simultaneously fit series of 1D spectra for each measurement to a decaying (or recovering) exponential function. In order to achieve this, we use a reference fit of the spectrum, also shown in Supplementary Figure 1, where the experimental, fitted, and residual spectra are displayed. This is performed with the INFOS software, which has been previously shown to accurately separate even heavily overlapped peaks, especially when fitting a series of spectra simultaneously.[4] This is critical, especially for chain resonances in POPC.

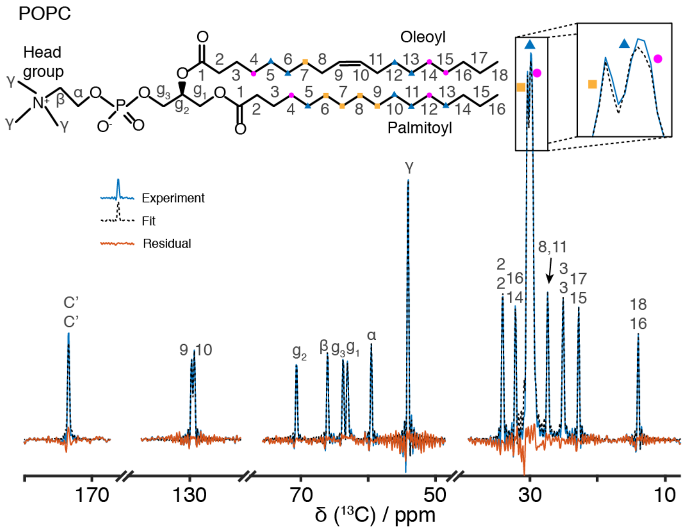

Supplementary Figure 1. Assignment and fit of resonances of ^{13}C POPC resonances. Experimental 1D spectrum is shown in blue, solid lines, a simulated spectrum that is fitted to the experiment is shown as a black dotted line, and the residual of this fit (fit-simulation) is plotted in red. Heavy overlap is observed around 30 ppm. We nonetheless fit three separate peaks, distinguished by a separated maximum at 30.3 ppm, and two peaks at 30.0 and 29.7 ppm. These were resolved with INFOS,[4] where differences in relaxation behavior simplify separation of resonances. These peaks are each assigned to several nuclei, as indicated on the POPC molecule at the top. Head group assignments were all fully resolved (α, β, γ, g_1, g_2, g_3), as were the double bonded nuclei found in the Oleoyl chain (9,10). Nuclei neighboring the double bond on the Oleoyl (8,11) are assigned to a single resonance. Remaining resonances were assigned to two nuclei, one for each chain (Oleoyl chain assignment shown above in the spectrum, Palmitoyl assignment below). These nuclei are in similar positions in the chains, either equidistant from the carbonyls, i.e. (2,2) and (3,3), or equidistant from the methyl groups, i.e. (16,14), (17,15), and (18,16), where the indices give the position in the Oleolyl and Palmitoyl groups, respectively.

1.2 Experiments

Four types of experiments were used in this study to experimentally characterize dynamics. These were ^{13}C T_1 relaxation, via saturation recovery, ^{1}H–^{13}C steady-state NOE, $T_{1\rho}$ relaxation (^{13}C transverse relaxation under spin-locking), and measurement of one-bond

^{1}H–^{13}C residual dipole couplings via DIPSHIFT. These are shown in Supplementary Figure 2, with experimental details given in Methods Table 1.

^{13}C T_1 experiments are performed by an initial saturation period of the ^{13}C magnetization, followed by a variable length recovery period (τ). Following the recovery period, a π/2-pulse is applied to the ^{13}C to read out the magnetization. Throughout the ^{13}C saturation and recovery period, ^{1}H magnetization is saturated, allowing us to decouple the evolution of ^{1}H magnetization from ^{13}C magnetization. Note that lipid samples are not labeled (1% ^{13}C from natural abundance). This prevents a significant influence from ^{13}C spin-diffusion.

Steady-state heteronuclear NOE was acquired by simply recycling the experiment with a long delay (10 s, at least 3.4x ^{13}C T_1), once with ^{1}H saturation and once without ^{1}H saturation. The ratio of these experiments yields the NOE enhancement ($\langle C_z^{SS} \rangle$), which is related to the ^{13}C R_1 and ^{1}H–^{13}C NOE rate constant (σ_{HC}) as follows:

$$\begin{aligned} \left\langle \mathrm{C}_z^{\mathrm{SS}} \right\rangle &= \left\langle \mathrm{C}_z \right\rangle_{\mathrm{eq}} \left(1 + \frac{\sigma_{\mathrm{HC}}}{R_{1,\mathrm{C}}} \frac{\gamma_{1\mathrm{H}}}{\gamma_{13\mathrm{C}}} \right) \\ \therefore \sigma_{\mathrm{HC}} &= \frac{\gamma_{13\mathrm{C}}}{\gamma_{1\mathrm{H}}} \left(\frac{\left\langle \mathrm{C}_z^{\mathrm{SS}} \right\rangle}{\left\langle \mathrm{C}_z \right\rangle_{\mathrm{eq}}} - 1 \right) R_{1,\mathrm{C}} \end{aligned} \tag{1}$$

^{13}C $T_{1\rho}$ experiments were acquired approximately on-resonance (see Methods), in order to avoid offset effects on the relaxation rate constants.[5] Experiments begin with a π/2-pulse on ^{13}C followed by variable length spin-lock, and acquisition directly after the spin-lock.

DIPSHIFT experiments were used to measure the one-bond ^{1}H–^{13}C dipole couplings.[6] Experiments begin with a ^{13}C π/2-pulse followed by homonuclear decoupling applied on ^{1}H for a variable fraction of one rotor period, followed by heteronuclear decoupling for the remainder of the rotor period. A ^{13}C π-pulse is applied and the ^{13}C chemical shift is allowed to refocus for one rotor period, followed by acquisition.

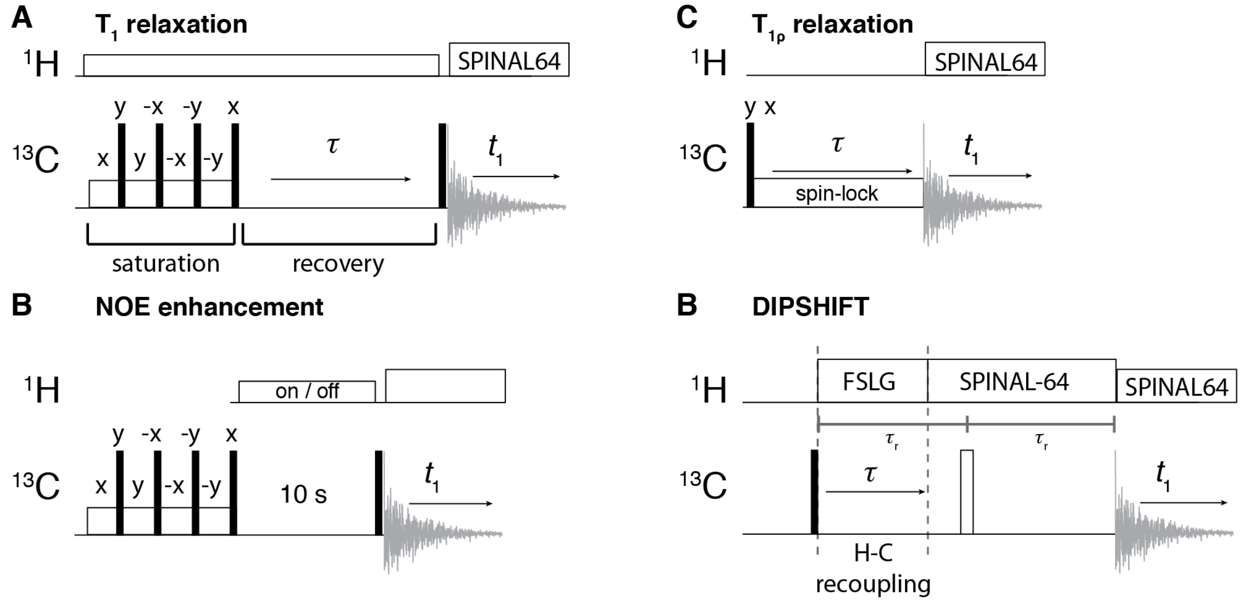

Supplementary Figure 2. Pulse sequences used in this study. **A** shows the T_1 saturation recovery experiment, where ^{13}C magnetization is destroyed and then recovers towards thermal equilibrium during τ. **B** shows the ^{1}H–^{13}C steady-state NOE measurement, where two experiments are compared, the first with ^{1}H saturation on, and the second with saturation off. **C** shows the $T_{1\rho}$ relaxation experiment, where magnetization is rotated from *z* to *x*, and spin-locked, to measure transverse magnetization decay during τ. **D** shows the DIPSHIFT experiment, where ^{13}C magnetization dephases under homonuclear decoupling within one rotor period, during time τ.

1.3 Extraction of rate constants

Spectra are processed in Bruker Topspin, using zero-filling to at least twice the number of time points, and exponential apodization. 18 separate resonances were identified. For each data series, a weighted sum of all experiments in the series were fitted (weighting set to maximize signal to noise, i.e. experiments with higher signal to noise are given higher weighting) using the INFOS “FitSpec” function in MATLAB,[4] in order to determine positions and linewidths of the 18 resonances (see Supplementary Figure 1). For relaxation measurements, the full series of spectra for each data set are fitted to a decaying exponential (decaying towards zero for $R_{1\rho}$ and towards some constant for R_1), using the INFOS “FitTrace” function, with variable amplitude and relaxation rate constant, but fixed position and linewidth (taken from the initial fit). An example fit is found in Supplementary Figure 3. NOE on and off signal intensities were found using the “FitSpec” function. DIPSHIFT spectra were analyzed separately, with amplitudes extracted for each separate spectrum. Then, for each peak, the extracted amplitudes are fitted against simulated DIPSHIFT curves. Curves are simulated for 200 order parameters ranging between 0 and 1 (referenced to a 21.5 kHz H–C dipole coupling), where frequency-switched Lee-Goldburg homonuclear decoupling is explicitly simulated,[7] and simulations include the correct number of ^{1}H for the given resonance. This allows one to correctly capture the dipole coupling scaling factor due to the decoupling, and to account for interference in dephasing due to

multiple [1]H. Simulations are performed using our own functions running in MATLAB (see MATLAB folder on on https://github.com/alsinmr/POPC_frames_archive).

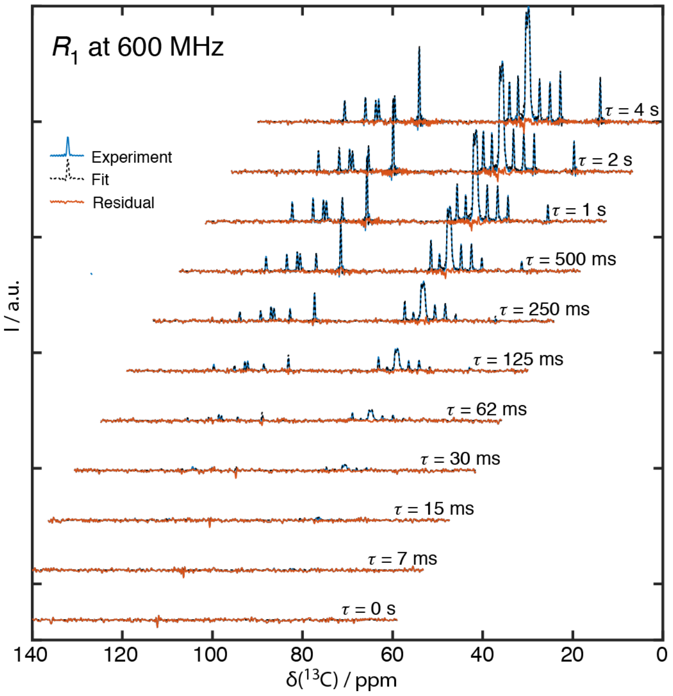

Supplementary Figure 3. Extraction of rate constants using the INFOS[4] FitTrace functionality. Here we show every second time point acquired for R_1 relaxation at 600 MHz, and truncate to show the range of the spectrum between 0 and 90 ppm (*x*-axis is correctly aligned for the spectrum at τ = 4 s, whereas prior spectra are shifted for better visibility). All spectra are fit simultaneously, using fixed positions and widths (determined from reference fit in Supplementary Figure 1), and variable amplitude and time constant (fit to the function $1-\exp(-\tau * R_1)$).

For all experiments, we obtain a standard deviation of the measured relaxation rate constant or order parameter using a simple bootstrap approach.[8] For DIPSHIFT and R_1/$R_{1\rho}$ measurements, we extract peak amplitudes from the series of spectra. Then, for each peak, we refit the corresponding curve 200 times, where we randomly resample the original data set. That is, suppose we have *N* data points for a given experiment and resonance (including multiple time points and also repetition of those time points). Then, we select *N* data points *with replacement* from the set, and refit the result. The standard deviation over parameters resulting from the 200 bootstrapped data sets is reported. For the NOE experiment, we extract error of the peak amplitudes using the “FitError” function of INFOS, and combined with the bootstrapped error for ^{13}C R_1, we apply the usual propagation-of-error rules to obtain an error for the NOE rate constant, although we note that this error results almost entirely from ^{13}C R_1 error, as the NOE peak heights may be very accurately extracted with INFOS. These errors are then used to determine the detector response error via linear propagation-of-error.

1.4 Detector analysis

Detectors can be thought of as a linear recombination of experimental rate constants. This is described in detail elsewhere,[9,10] but a brief review of the work and some updates to computational methods is merited here. We first, assume that the correlation function of motion (either experimental or MD-derived) is a sum of decaying exponential terms, such that

$$\begin{aligned} &C(t) = S^2 + (1-S^2)\int_{-\infty}^{\infty} \theta(z)\exp(-t/(10^z \cdot 1\text{ s}))dz \\ &0 \le S^2 \le 1, \quad \int_{-\infty}^{\infty} \theta(z)dz = 1 \end{aligned} \quad . \qquad (2)$$

Then, the correlation function always begins at 1 and plateaus at S^2. The rate of decay may be described by one or more correlation times, where $\theta(z)$ indicates decay is distributed as a function of correlation time (z is the log-correlation, $z = \log_{10}(\tau_c / \text{s})$). Then, a relaxation rate constant (indexed by ζ) is equal to

$$R_\zeta^{(\theta,S)} = (1-S^2)\int_{-\infty}^{\infty} \theta(z)R_\zeta(z)dz, \qquad (3)$$

where $R_\zeta(z)$ is the sensitivity of experiment ζ. The sensitivity is the relaxation rate constant's dependence on correlation time. That is, if a correlation function has mono-exponential decay with rate constant τ_c and decays completely to 0 ($S^2 = 0$), then the resulting rate constant for experiment ζ is $R_\zeta(z)$ for $z = \log_{10}(\tau_c / \text{s})$. In this case, the integral in (3) then adds up the scaled contributions for all correlation times in a multi-exponential correlation function, where the scaled contribution is the product of the amplitude of decay for that correlation time and the sensitivity of the experiment at that correlation time, $R_\zeta(z)$. Examples of $R_\zeta(z)$ are given in Supplementary Figure 4A (top). Then, the basic concept behind detectors is that linear combinations of rate constants can be created to generate detectors that are sensitive to specific timescale windows. Suppose we take a sum of experimental rate constants, which we will define as

$$
\begin{aligned}
\rho_n^{(\theta,S)} &= \sum_{\zeta} a_{n,\zeta} R_{\zeta}^{(\theta,S)} \\
&= \sum_{\zeta} a_{n,\zeta} (1-S^2) \int_{-\infty}^{\infty} \theta(z) R_{\zeta}(z) dz \\
&= (1-S^2) \int_{-\infty}^{\infty} \theta(z) \underbrace{\sum_{\zeta} a_{n,\zeta} R_{\zeta}(z)}_{\rho_n(z)} dz \text{'} \\
\rho_n^{(\theta,S)} &= (1-S^2) \int_{-\infty}^{\infty} \theta(z) \rho_n(z) dz
\end{aligned}
\tag{4}
$$

The linear combination yields a new parameter with a new relationship to the distribution of correlation times of motion, $(1-S^2)\theta(z)$. The relationship is defined by the sensitivity of the detector, $\rho_n(z) = \Sigma_{\zeta} a_{n,\zeta} R_{\zeta}(z)$. Then, a detector response, $\rho_n^{(\theta,S)}$, quantifies the amplitude of motion having correlation times in the window defined by the detector sensitivity. Specifically, via computation of $\rho_n^{(\theta,S)}$, we acquire the overlap integral of the distribution of correlation times of motion, $(1-S^2)\theta(z)$, with the detector sensitivity, $\rho_n(z)$. For detector responses to be easily interpreted, one must then optimize the weighting of the experiments $(a_{n,\zeta})$ in the data set to obtain narrow, well-separated sensitivities. An example of this linear recombination is found in Supplementary Figure 4, where experimental sensitivities are weighted and summed in B(top) to yield $\rho_1(z)$ in C (orange, bold). The same linear combination is applied to experimentally measured rate constants (see first line of Supplementary Equation (4)), where this is shown in Supplementary Figure 4E(top) with the resulting sum in F. One observes that the detector responses in Supplementary Figure 4F are the result of the sum of the amplitudes for each of three correlation motions in the distribution in F ($(1-S^2)\theta(z)$, left axis) multiplied by the sensitivity of the corresponding detector at the each motion's correlation time. Note that in this simplified example, we neglect the role of variance of each measurement in optimization and linear recombination, where details are provided previously.[9] A similar example was provided in detail in a recent paper on application of detectors to solution-state NMR.[11]

MD simulated data may, in fact, be treated as equivalent to NMR data. Compare equations (2) and (3): excepting for the offset of S^2 appearing in $C(t)$, we can say that whereas $R_{\zeta}(z)$ is the sensitivity of the relaxation rate constant, $\exp(-t/(10^z \cdot 1\,\mathrm{s}))$ is the sensitivity of time point, t, in the correlation function. An example of these sensitivities is shown in Supplementary Figure 4A (bottom), where t is fixed, but z is swept. Then, a linear

recombination of time points of the MD-derived correlation functions may be used to generate detectors for the MD simulation. Linear recombination of sensitivities of a few time points of a correlation function are shown in Supplementary Figure 4B (bottom), to yield $\rho_1(z)$ in C (black, bold, dashed line). This line is nearly identical to the experimentally derived detector sensitivity (orange, bold). This approach contrasts to our prior work with MD simulation, where we first performed an inverse Laplace transform on MD-derived correlation functions, followed by multiplication with the detector sensitivity function, and integration.[12]

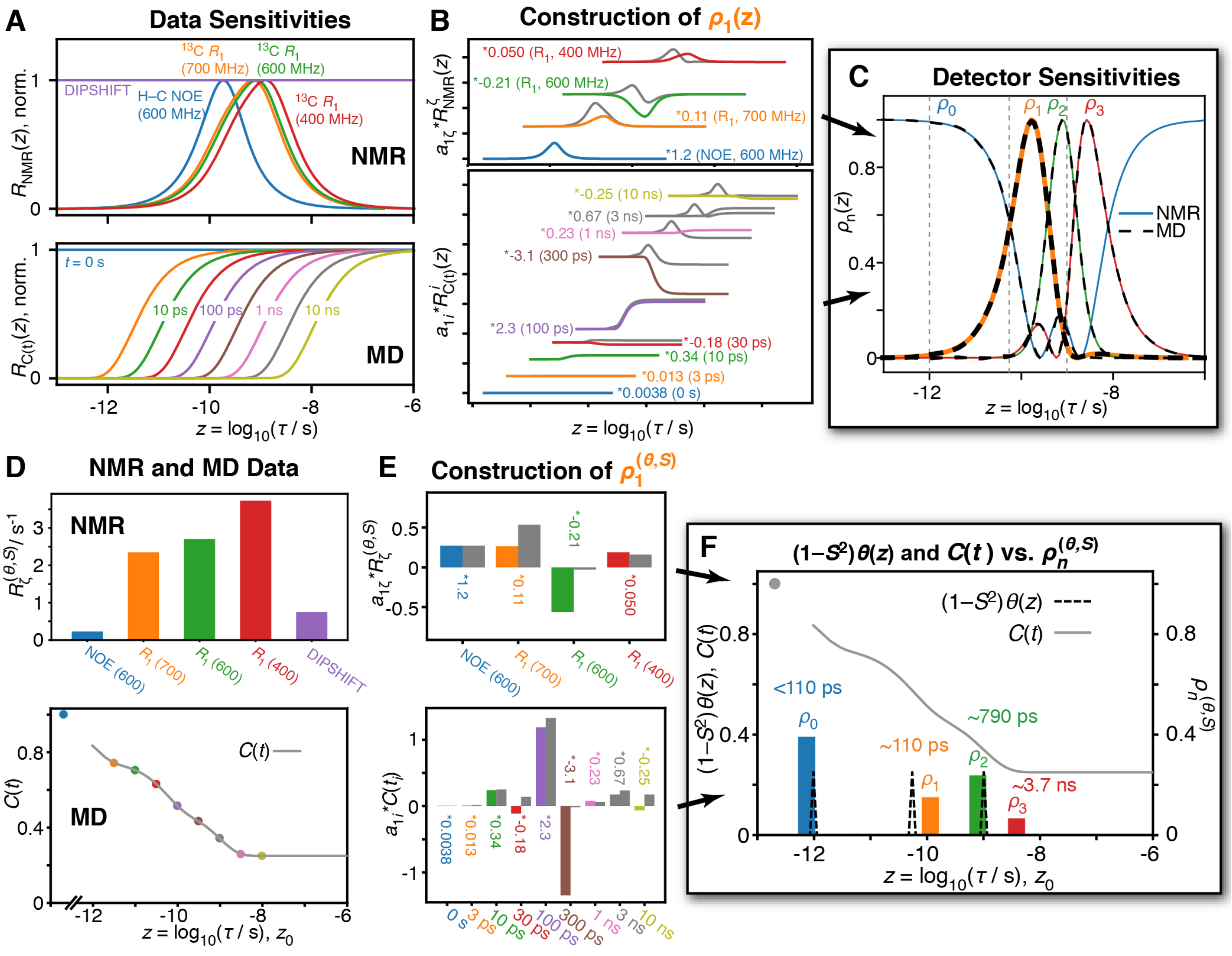

Supplementary Figure 4. Detector construction from NMR and MD simulation. **A** shows the sensitivity windows for five NMR experiments (^{13}C R_1 at 400, 600, 700 MHz, ^{1}H–^{13}C NOE at 600 MHz, and DIPSHIFT) and for 9 time points extracted from MD (0 s, 3 ps, 10 ps, 30 ps, …10 ns). **B** shows the weighted sensitivities for both NMR relaxation (excluding DIPSHIFT) and MD time points, where the sum of the weighted sensitivities yields detector sensitivity $\rho_1(z)$ in **C**. In **B**, grey lines show the cumulative sums of all sensitivities at and below the grey line. Sums of the weighted sensitivities in **B** yield $\rho_1(z)$ shown in **C**. **C** compares four detector sensitivities derived from NMR sensitivities (color) and MD (black, dashed). ρ_1 is shown in bold, since this is the sensitivity that is obtained in **B**. **D** shows experimental and simulated data, obtained for a distribution having three correlation times (distribution in **F**, black, dashed lines, left axis). **E** shows the linear recombination of those data points to yield the detector response for ρ_1, where the weighting matches the linear combination in **B** (see Supplementary Equation (4)). **F** shows the resulting detector responses (colored bars, right axis). For comparison, the distribution of motion ($(1-S^2)\theta(z)$, black, dashed lines) is also shown, along with the correlation function (left axis). Detector responses are largest when a motion falls near the center of the detector, or equivalently, when the correlation function is changing rapidly near the detector center.

A few additional considerations must be taken for analysis of MD-derived correlation functions with detectors. First, we must deal with the offset term, S^2. This term collects non-decaying components of the correlation function. Practically, however, there is no difference between non-decaying components and very slowly decaying components (for example, any motion at least ~5 times slower than the length of our MD simulations, so about 42 μs, will not appear to decay during the simulation). Then, we simply neglect S^2 when analyzing MD-derived correlation functions with detectors, and allow its contribution to be part of the distribution, $\theta(z)$, for z corresponding to arbitrarily long correlation times. Furthermore, we require the *relative* standard deviations of the various time points in a correlation function when optimizing detectors. We assume that the standard deviation is proportional to $m^{-1/2}$, where m is the number of pairs of time points used to calculate that element of the correlation function. m is given by N–n, where N is the total number of time points in the MD simulation, and n is the index of the time point of the correlation function. Note that the first element of the correlation function is *exactly* 1 in all cases. To deal with this numerically, we set its standard deviation to $1\text{x}10^{-7}$, which is sufficiently small to force a nearly exact fit of this time point, but sufficiently large to avoid dynamic range problems in our calculations.

1.5 Experimental and Simulated Detector Optimization

Detector optimization is performed as described previously:[10] we take the sensitivities of all experiments, normalized by their corresponding standard deviations, collected in matrix **M**. We then perform a singular value decomposition of **M**, taking the largest n singular values and corresponding vectors, such that

$$\mathbf{M} \approx \widetilde{\mathbf{M}} = \mathbf{U_t} \cdot \Sigma_t \cdot \mathbf{V}_t' , \tag{5}$$

$\widetilde{\mathbf{M}}$ is an approximation of **M**, and in fact is the best approximation of **M** possible via linear combination of t vectors. Columns of $\mathbf{V}_t$ are then linearly recombined to form detectors. By first selecting the largest singular values, we improve the data fit and minimize error of the resulting detectors. Large choices of n will yield higher resolution detectors, but more error.

Linear recombination of the columns of $\mathbf{V}_t$ may be optimized to either match some target function, or to yield detectors with minimal width and overlap. The former case is used when comparing MD results to NMR results: we already have NMR sensitivities, and so we optimize MD sensitivities to match the NMR sensitivities. This may be achieved with the 'lstsq' algorithm (linear least squares) in NumPy's linear algebra module.[13] Note that we use the 15 largest singular values when using this approach. However, we must use the latter case when optimizing the experimental detectors since we do not have an obvious

target function. In this case, we sweep through an array of 200 correlation times (log-spaced from 10^{-14} to 10^{-3} s), and at each correlation function, we attempt to optimize a detector sensitivity that is equal to 1 at the current correlation time, and minimized, but non-negative elsewhere. This is achieved with the 'linprog' algorithm in SciPy's optimization module.[14] Then, if *n* singular values are used, we find that for exactly *n* correlation times, the *maximum* of the sensitivity is found at the same correlation time where the sensitivity is forced to equal 1. We take the detectors from these *n* sensitivities. For experimental analysis, we take *n* equal to 6. When analyzing MD data without matching sensitivities to experiment, we take *n* equal to 6 or 9 (6: Supplementary Figure 10, 9: Supplementary Figure 8, Supplementary Figure 12, Supplementary Figure 15,).

1.6 Experimental data fits

For each resonance in Supplementary Figure 1, 8 experiments are analyzed (6 for carbonyls) using detector analysis. Each different spin-system requires a separate matrix **r**, which contains the detection vectors.[9] The corresponding matrices are given here (Supplementary Table 1–Supplementary Table 4), for ^{13}C having one bonded ^{1}H, two bonded ^{1}H, three bonded ^{1}H, or no bonded ^{1}H, but large chemical shift anisotropy (carbonyls). Then, for a set of experimental rate constants ($R_\zeta^{\text{exp.}}$) having standard deviations ($\sigma(R_\zeta)$), we minimize

$$\min \sum_\zeta \sum_n \frac{\left(R_\zeta^{\text{exp.}} - [\mathbf{r}]_{\zeta,n}\rho_n^{(\theta,S)}\right)^2}{\sigma(R_\zeta)^2} \tag{6}$$

where the $\rho_n^{(\theta,S)}$ are varied (bound between $\min(\rho_n(z))$ and $\max(\rho_n(z))=1$), using the 'lstsq' algorithm of NumPy's linear algebra module.[13]

The resulting data fits are shown in Supplementary Figure 5, and tabulated in Supplementary Table 5. Note that for carbonyls, no DIPSHIFT or NOE data is available due to the lack of a bonded ^{1}H. For DIPSHIFT, we note that S^2 is already very small, and so we simply set to the mean MD-simulated value (0.0365), with minimal impact on the results (slightly lowering $\rho_0^{(\theta,S)}$ vs. if we just set the value to 0). For the NOE, we exclude this data, resulting in one fewer detector, and modified detector sensitivities for C', which are shown in Supplementary Figure 6. Note when validating C' motion with MD, we use the modified sensitivities to obtain a more meaningful comparison.

Supplementary Table 1: Detection vectors for fitting carbonyls

	$\vec{r}_0$ / s^{-1}	$\vec{r}_2$ / s^{-1}	$\vec{r}_3$ / s^{-1}	$\vec{r}_4$ / s^{-1}	$\vec{r}_5$ / s^{-1}
$\sigma_{HC,600}$	0.000	-1.236e-11	1.153e-11	-2.111e-11	3.219e-11
$R_{1,700}$	0.000	3.247	1.822	0.0002750	-0.0004210
$R_{1,600}$	0.000	2.482	1.907	-7.721e-6	-0.0002439
$R_{1,400}$	0.000	1.064	1.835	0.0004295	-0.0002748
$R_{1\rho,22}$	0.000	-0.5863	9.277	1.598e4	2117
$R_{1\rho,12}$	0.000	1.074	0.4852	1.712e4	3.828e4
$R_{1\rho,7}$	0.000	-14.31	27.88	1.179e4	7.639e4
$(1–S^2)$	1.000	0.6911	0.6290	0.6890	0.8268

Other parameters: $\Delta\sigma$=232.5 ppm

Supplementary Table 2: Detection vectors for fitting carbons with one ^{1}H

	$\vec{r}_0$ / s^{-1}	$\vec{r}_1$ / s^{-1}	$\vec{r}_2$ / s^{-1}	$\vec{r}_3$ / s^{-1}	$\vec{r}_4$ / s^{-1}	$\vec{r}_5$ / s^{-1}
$\sigma_{HC,600}$	0.000	0.9979	0.2669	0.04406	-3.467e-5	-6.237e-5
$R_{1,700}$	0.000	1.980	3.258	1.218	-3.024e-5	-0.0006976
$R_{1,600}$	0.000	2.019	3.838	1.716	-0.0001457	-0.0008049
$R_{1,400}$	0.000	1.993	5.464	3.866	0.0004401	-0.001526
$R_{1\rho,22}$	0.000	0.9854	2.025	10.31	1.591e4	2125
$R_{1\rho,12}$	0.000	-0.8422	4.559	-2.156	1.734e4	3.813e+4
$R_{1\rho,7}$	0.000	-1.122	-10.39	26.51	1.236e4	7.610e+4
$(1–S^2)$	1.000	0.7611	0.6583	0.7815	0.7033	0.8337

Other parameters: δ_{HC}=46640 Hz

Supplementary Table 3: Detection vectors for fitting carbons with two ^{1}H

	$\vec{r}_0$ / s^{-1}	$\vec{r}_1$ / s^{-1}	$\vec{r}_2$ / s^{-1}	$\vec{r}_3$ / s^{-1}	$\vec{r}_4$ / s^{-1}	$\vec{r}_5$ / s^{-1}
$\sigma_{HC,600}$	0.000	0.9972	0.2667	0.04404	-3.465e-5	-6.241e-5
$R_{1,700}$	0.000	3.957	6.510	2.434	-6.259e-5	-0.001395
$R_{1,600}$	0.000	4.034	7.671	3.429	-0.0002884	-0.001612
$R_{1,400}$	0.000	3.984	10.92	7.729	0.0008799	-0.003052
$R_{1\rho,22}$	0.000	1.977	4.020	20.64	3.181e4	4253
$R_{1\rho,12}$	0.000	-1.803	9.571	-4.756	3.469e4	7.628e4
$R_{1\rho,7}$	0.000	-1.919	-22.01	54.21	2.472e4	1.523e5
$(1–S^2)$	1.000	0.7639	0.6501	0.7864	0.7043	0.8347

Other parameters: δ_{HC}=46640 Hz

Supplementary Table 4: Detection vectors for fitting carbons with three ^{1}H

	$\vec{r}_0$ / s^{-1}	$\vec{r}_1$ / s^{-1}	$\vec{r}_2$ / s^{-1}	$\vec{r}_3$ / s^{-1}	$\vec{r}_4$ / s^{-1}	$\vec{r}_5$ / s^{-1}
$\sigma_{HC,600}$	0.000	0.9977	0.2669	0.04406	-3.449e-5	-6.250e-5
$R_{1,700}$	0.000	5.938	9.772	3.653	-9.648e-5	-0.002091
$R_{1,600}$	0.000	6.055	11.51	5.146	-0.0004335	-0.002418
$R_{1,400}$	0.000	5.979	16.39	11.60	0.001319	-0.004582
$R_{1\rho,22}$	0.000	2.977	5.995	31.02	4.763e4	6384.
$R_{1\rho,12}$	0.000	-2.884	14.99	-7.922	5.198e4	1.145e5
$R_{1\rho,7}$	0.000	-2.398	-34.74	83.46	3.688e4	2.286e5
$(1–S^2)$	0.9999	0.7608	0.6595	0.7813	0.6969	0.8311

Other parameters: δ_{HC}=46640 Hz

Supplementary Table 5: Experimental relaxation rates and fits with detectors

		$\sigma_{HC,600}$	$R_{1,700}$	$R_{1,600}$	$R_{1,400}$	$R_{1\rho,22}$	$R_{1\rho,12}$	$R_{1\rho,7}$	S^2
γ	$R_{exp.}$	0.258	1.63	1.88	2.02	2.73	5.62	5.81	0.00
	$\sigma_{exp.}$	0.0129	0.0768	0.0901	0.0569	0.238	0.504	0.648	0.00140
	R_{fit}	0.245	1.76	1.85	2.01	2.81	5.11	6.22	0.00
β	$R_{exp.}$	0.322	2.02	2.20	4.58	6.06	10.3	14.5	0.00360
	$\sigma_{exp.}$	0.0308	0.122	0.200	0.213	0.605	0.660	1.48	0.00110
	R_{fit}	0.262	2.34	2.69	3.77	6.34	9.85	15.6	0.00360
α	$R_{exp.}$	0.323	1.88	2.41	3.37	7.94	15.6	17.5	0.00310
	$\sigma_{exp.}$	0.0164	0.0997	0.127	0.100	0.524	1.37	1.82	0.00200
	R_{fit}	0.306	2.17	2.44	3.21	8.14	13.7	19.2	0.00310
g_3	$R_{exp.}$	0.340	4.58	4.58	7.17	27.0	47.6	34.5	0.0511
	$\sigma_{exp.}$	0.0146	0.635	0.221	0.182	2.43	4.78	5.51	0.00170
	R_{fit}	0.337	4.30	4.99	7.02	28.0	42.3	37.9	0.0511
g_2	$R_{exp.}$	0.314	2.02	2.20	3.88	23.8	52.6	83.3	0.0365
	$\sigma_{exp.}$	0.0140	0.125	0.108	0.164	1.49	4.97	10.9	0.00240
	R_{fit}	0.314	1.91	2.32	3.81	23.9	50.6	88.1	0.0365
g_1	$R_{exp.}$	0.323	3.37	3.88	7.17	43.5	62.5	66.7	0.0212
	$\sigma_{exp.}$	0.0198	0.152	0.217	0.231	2.00	3.86	6.29	0.00140
	R_{fit}	0.312	3.54	4.24	6.72	44.9	55.4	75.7	0.0212
C’	$R_{exp.}$	–	0.922	1.24	0.570	12.3	20.4	22.2	*0.0365
	$\sigma_{exp.}$	–	0.0514	0.140	0.0204	0.665	1.04	1.77	*0.0100
	R_{fit}	–	0.989	0.855	0.561	12.8	18.8	24.4	*2.0e-15
2,2	$R_{exp.}$	0.359	2.02	2.97	3.88	24.4	38.5	40.0	0.0259
	$\sigma_{exp.}$	0.0147	0.0979	0.128	0.100	0.895	2.34	2.41	0.00190
	R_{fit}	0.345	2.42	2.74	3.77	24.9	33.5	42.5	0.0259
3,3	$R_{exp.}$	0.346	1.88	2.41	2.97	19.2	30.3	30.3	0.0404
	$\sigma_{exp.}$	0.0114	0.100	0.0857	0.0643	0.745	2.24	2.37	0.00140
	R_{fit}	0.342	2.12	2.34	2.95	19.4	27.8	31.6	0.0404
●	$R_{exp.}$	0.264	1.18	1.63	1.88	10.1	18.2	22.7	0.0243
	$\sigma_{exp.}$	0.00320	0.0466	0.0196	0.0249	0.737	2.12	2.32	0.00230
	R_{fit}	0.263	1.46	1.58	1.89	10.1	17.8	22.9	0.0243
▲	$R_{exp.}$	0.276	1.18	1.75	2.02	17.2	34.5	26.3	0.0309
	$\sigma_{exp.}$	0.00550	0.0533	0.0365	0.0383	0.697	2.05	1.89	0.00230
	R_{fit}	0.272	1.53	1.66	2.00	17.9	26.4	29.6	0.0309
■	$R_{exp.}$	0.263	1.24	1.63	1.88	20.4	31.2	33.3	0.0445
	$\sigma_{exp.}$	0.00430	0.0790	0.0255	0.0437	0.859	2.54	2.14	0.00230
	R_{fit}	0.263	1.49	1.61	1.89	20.4	31.1	33.4	0.0445
8,11 oleoyl	$R_{exp.}$	0.283	1.37	1.88	2.41	8.70	19.6	21.3	0.0134
	$\sigma_{exp.}$	0.00930	0.0778	0.0568	0.0420	0.436	1.12	1.57	0.00260
	R_{fit}	0.272	1.71	1.88	2.36	8.96	17.2	23.5	0.0134
9 oleoyl	$R_{exp.}$	0.501	1.45	1.88	1.75	11.0	38.5	32.3	0.00910
	$\sigma_{exp.}$	0.0395	0.0827	0.128	0.0364	0.753	4.06	3.74	0.00160
	R_{fit}	0.520	1.44	1.54	1.77	11.3	26.6	37.1	0.00910
10 oleoyl	$R_{exp.}$	0.653	1.53	2.41	1.88	5.24	11.8	15.6	0.00360
	$\sigma_{exp.}$	0.0359	0.0657	0.140	0.0549	0.535	0.787	0.863	0.00150
	R_{fit}	0.657	1.63	1.73	1.91	5.27	11.7	15.7	0.00360
14,16	$R_{exp.}$	0.137	0.534	0.805	1.30	8.00	14.3	16.9	0.0145
	$\sigma_{exp.}$	0.00440	0.0348	0.0246	0.0446	0.342	0.916	1.03	0.00290
	R_{fit}	0.131	0.710	0.804	1.17	8.34	10.9	19.0	0.0145
15,17	$R_{exp.}$	0.110	0.311	0.583	0.922	4.95	11.0	12.7	0.0101
	$\sigma_{exp.}$	0.00570	0.0451	0.0323	0.0302	0.218	0.700	0.952	0.00130
	R_{fit}	0.0996	0.554	0.619	0.849	5.12	8.58	14.8	0.0101
16,18	$R_{exp.}$	0.0491	0.293	0.341	1.03	2.00	4.50	5.26	0.00160
	$\sigma_{exp.}$	0.00460	0.0329	0.0341	0.0611	0.275	0.339	0.510	0.00190
	R_{fit}	0.0373	0.401	0.455	0.626	2.33	3.81	6.02	0.00160

*Data taken from MD. Measured S^2 are all nearly zero, where deviation to ~0.02 compared to surrounding residues is unlikely to make a significant impact on results (changes ρ_0 slightly)

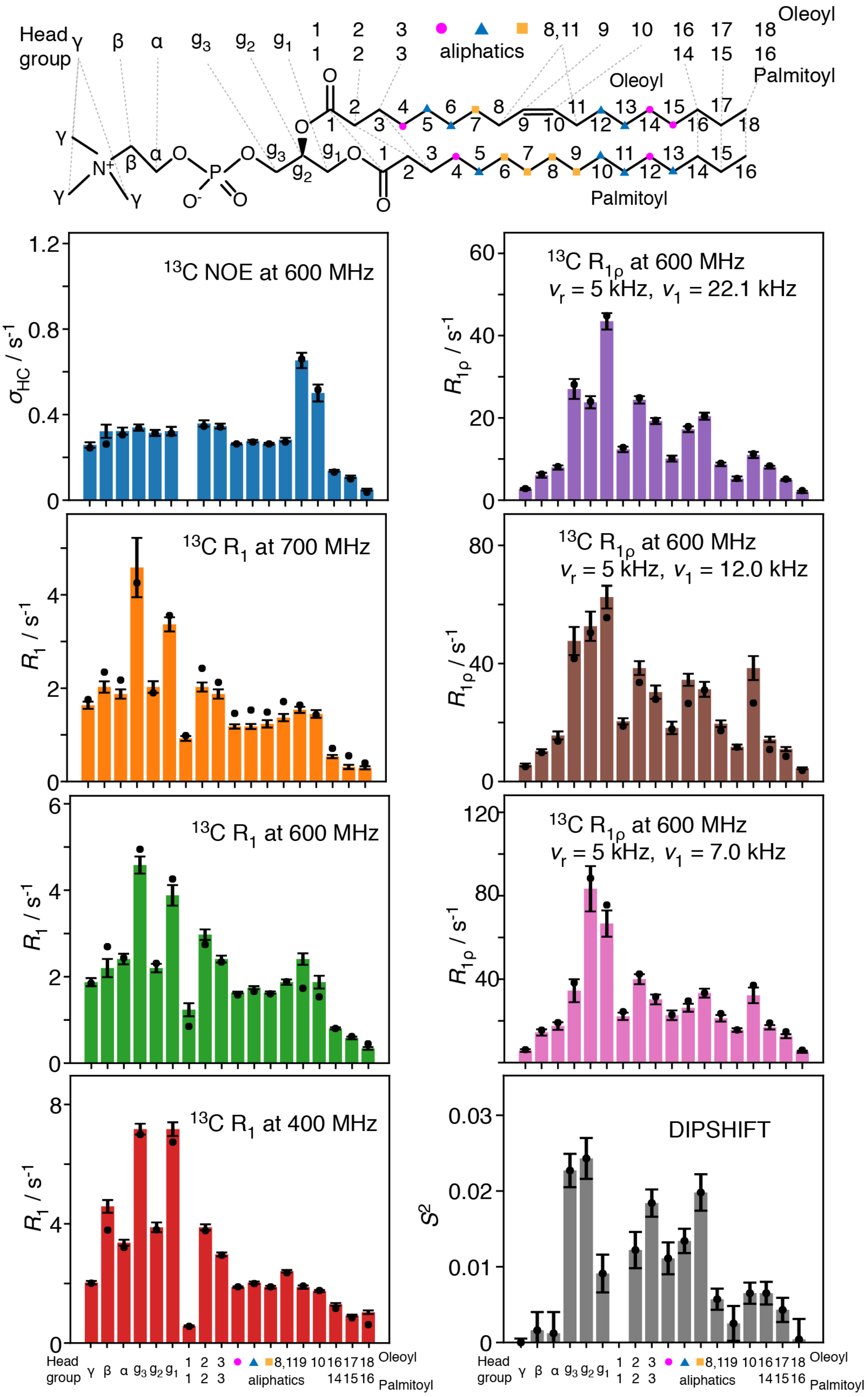

Supplementary Figure 5. Fits of experimental data. At top, we show the positions of each resonance on the POPC molecule. In each plot, we show the experimentally measured values as colored bars, with black error bars indicating $\pm\sigma$ for that measurement. σ is the standard deviation of bootstrap samples, as described in Supplementary Note 1.3. Black scatter points indicate the values obtained using detector fitting. Source data are provided as a Source Data File.

1.7 Additional Information on Detector Analysis

In our experimental data analysis, we have included relaxation from carbonyls in POPC. Because these carbons have no bonded proton, neither the DIPSHIFT or NOE experiments can be used. While we estimate the DIPSHIFT result based on simulation (vida supra), we must omit the NOE data, resulting in one fewer detector for carbonyls. The resulting detectors are compared to those obtained for other positions in Supplementary Figure 6. One sees that $\rho_0(z)$ obtained for C' (black, dashed) extends to longer correlation times than for other positions (color), and $\rho_1(z)$ is missing. $\rho_2(z)$ and $\rho_3(z)$ are shifted to slightly shorter correlation times, whereas $\rho_4(z)$ and $\rho_5(z)$ are practically unchanged.

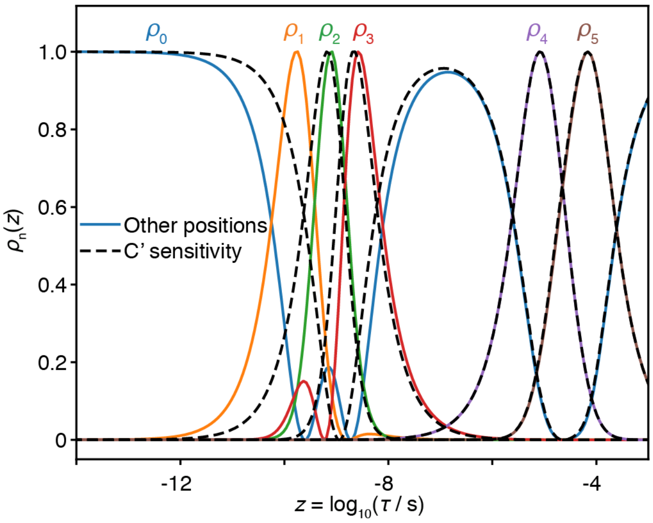

Supplementary Figure 6. Comparison of C'-sensitivity to sensitivity at other positions. Colored lines plot six sensitivities obtained for all positions of POPC except the C'. Sensitivities of C' are plotted as black, dashed lines. Missing NOE data requires us to omit one detector for analysis of C' relaxation, resulting in ρ_1 being absent for C' analysis. Furthermore, $\rho_0(z)$ is shifted to longer correlation times, and $\rho_2(z)$ and $\rho_3(z)$ are shifted to slightly shorter correlation times.

A second challenge in the experimental analysis is that $\rho_0(z)$ contains multiple regions of non-zero sensitivity: one region capturing all motion faster than about 110 ps, and a second window capturing motion slower than ~3.7 ns but faster than ~6.3 μs (a third window appears around 1 ms, but the small experimental detector responses for ρ_4 and ρ_5 indicate that it is unlikely to contribute significantly to $\rho_0^{(\theta,S)}$). Given the initial agreement between experiment and simulation, we may use MD simulation to determine how important those multiple windows are. In Supplementary Figure 7, we generate two detector windows and corresponding detector responses. The first window in A corresponds to the ρ_0 sensitivity for MD in main text Fig. 1A (blue, solid line), but the second window sets sensitivities to approximately zero for correlation times longer than 1.8 ns (blue, dashed

lines). The corresponding detector responses are shown in B, where significant differences are only seen in the glycerol and C' carbons. Therefore, for all other positions, $\rho_0^{(\theta,S)}$ characterizes only motion faster than ~110 ps. For subsequent MD analysis, we therefore use the modified detector, for which $\rho_0(z)$ is approximately zero for correlation times longer than 1.8 ns.

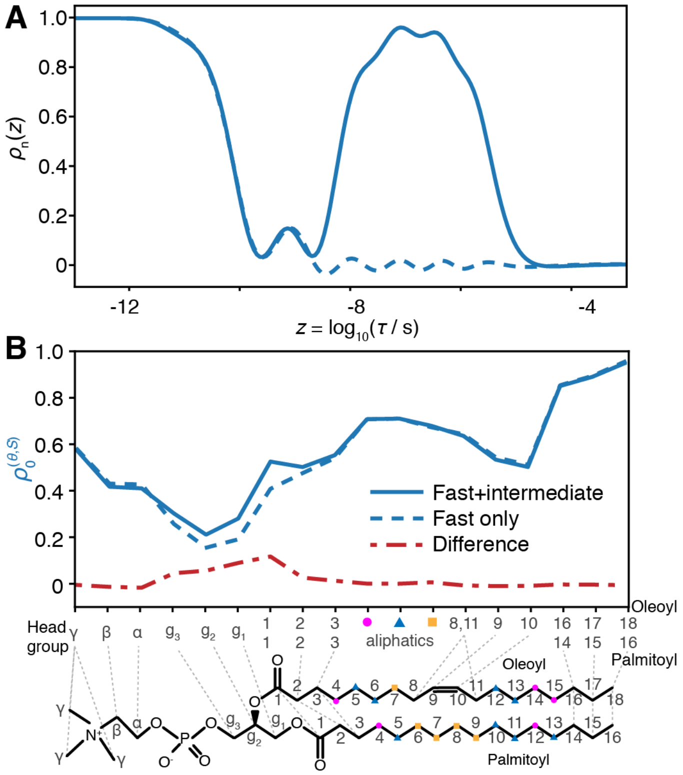

Supplementary Figure 7. Influence of ns–μs range motion on $\rho_0^{(\theta,S)}$ in MD simulation. In **A**, we plot the sensitivity of ρ_0 used in main text Fig. 1 (solid line), and an additional, modified $\rho_0(z)$, where correlation times longer than 1.8 ns are set to approximately 0 (dashed line). **B** shows the corresponding ρ_0 detector responses, obtained from analysis of MD data for the two sensitivities (blue, dashed line indicates the modified sensitivity). Red line shows the difference, where we see that slow motions ($\tau_c > 1.8$ ns) contributes only marginally to motion in the glycerol backbone and to C' carbons. Source data for **B** are provided as a Source Data File.

1.8 Comparison of MD simulations

Overall dynamics of POPC in a membrane depends not only on the local environment, but also on collective motion of the membrane. This motion will necessarily depend on the size of the simulation, where correlation length of wave-like motions will have an upper bound equal to the MD box size. Then, we want to verify that this type of motion is not strongly influencing timescales for which we make comparisons to NMR data. Therefore, three simulations were run: one containing 256 POPC molecules (8.4 μs), one containing 1024 molecules (9.5 μs), and one containing 4096 molecules (2.0 μs). A detector analysis in Supplementary Figure 8 is performed on the three simulations, where detectors are

optimized to match for each simulation. Then, we see that small deviations in detector responses appear near 75 ns, and become larger near 300 ns and 1.5 μs. Considering that we compare MD to experimental detectors only out to about 3.7 ns, we should not see discrepancies due to the size of the system, such that all other analyses in this study rely only on simulations with 256 POPC molecules.

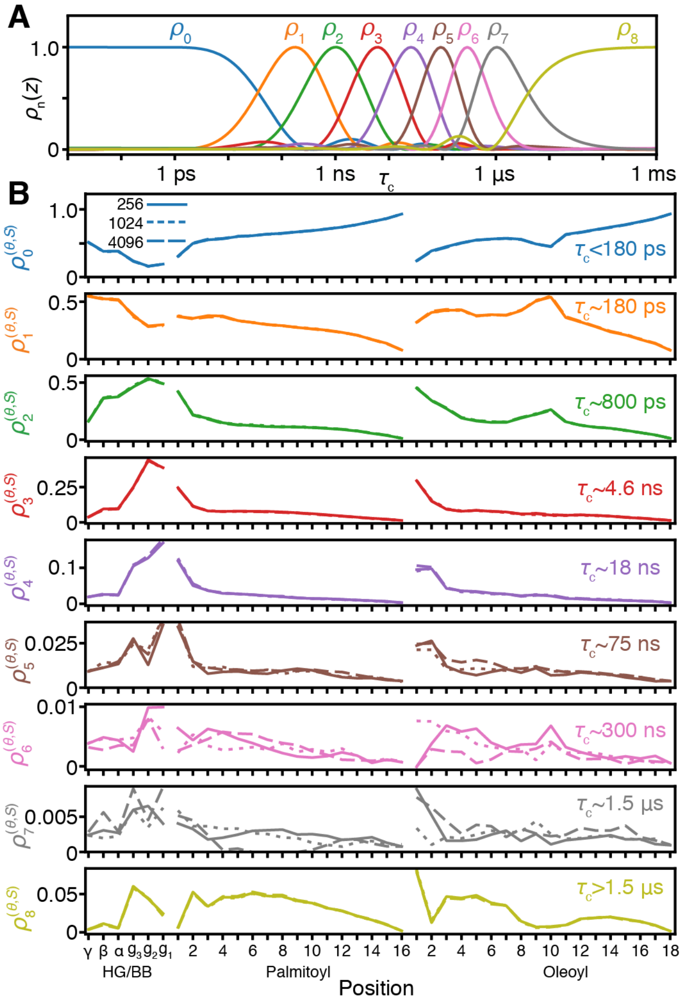

Supplementary Figure 8. Comparison of detector responses for MD runs with 256, 1024, and 4096 copies of the POPC molecule. Nine detectors are optimized for comparison (**A**), where each plot in **B** shows the detector responses for one of the nine detectors for each of the three simulations (solid lines: 256, dotted lines: 1024, dashed lines: 4096). Source data are provided as a Source Data File.

Supplementary Note 2: Frame analysis

In order to separate motions into components, one must select frame definitions, and test them to determine if the resulting motions in the frame and motion of the frame are statistically independent, and sufficiently timescale-separated. One may perform this frame screening by simply computing the total correlation function directly, and computing the product of the individual correlation functions. Good agreement indicates a good choice of frames. Note that if we assume that the individual correlation functions are multi-exponential, as in Supplementary Equation (2), then the product must also be multi-

exponential. We demonstrate this for a product of two correlation functions, but the result may be easily iterated over multiple correlation functions.

$$
\begin{aligned}
C(t) &= C^1(t)\cdot C^2(t) \\
&= \left[S_1^2+(1-S_1^2)\int_{-\infty}^{\infty}\theta_1(z)e^{-t/10^z\cdot s}\,dz\right]\left[S_2^2+(1-S_2^2)\int_{-\infty}^{\infty}\theta_2(z)e^{-t/10^z\cdot s}\,dz\right] \\
&= S_1^2S_2^2+S_2^2(1-S_1^2)\int_{-\infty}^{\infty}\theta_1(z)e^{-t/10^z\cdot s}\,dz+S_1^2(1-S_2^2)\int_{-\infty}^{\infty}\theta_2(z)e^{-t/10^z\cdot s}\,dz+ \\
&\qquad (1-S_1^2)(1-S_2^2)\int_{-\infty}^{\infty}\int_{-\infty}^{\infty}dz_1\,dz_2\theta_1(z_1)\theta_2(z_2)\underbrace{e^{-t*\left(10^{-z_1}\cdot s+10^{-z_2}\cdot s\right)}}_{e^{-t/10^{-z_{\mathrm{eff}}}\cdot s}} \\
&= S^2+(1-S^2)\int_{-\infty}^{\infty}\theta(z)e^{-t/10^z\cdot s}\,dz
\end{aligned}
\tag{7}
$$

One sees that the total correlation function resulting from the product of two multi-exponential correlation functions is indeed itself multi-exponential. The resulting correlation function has an offset term, $S^2=S_1^2S_2^2$, includes the distribution corresponding to the first correlation function, scaled by S_2^2 and the second correlation function, scaled by S_1^2, and finally a double integral containing effective correlation times resulting from the product of two exponential terms coming from each of the correlation functions. Note that an explicit expression for $(1-S^2)\theta(z)$ may be obtained in analogy to the previous result from Smith et al.[10]

$$
\begin{aligned}
(1-S^2)\theta(z) &= S_2^2(1-S_1^2)\theta_1(z)+S_1^2(1-S_2^2)\theta_2(z)+ \\
&\qquad (1-S_1^2)(1-S_2^2)\int_{z}^{\infty}\theta_1(z_1)\theta_2(z+z_1-\log_{10}(10^{z_1}-10^{z}))\frac{10^{z_1}}{10^{z_1}-10^{z}}dz_1
\end{aligned}
\tag{8}
$$

Each of the two distributions contributes to the total distribution, scaled by the order parameter of the other distribution. Additionally, effective correlation times arise from the product of the decaying parts of both correlation functions, yielding the integral in the second line.

Now that we see how a product of multi-exponential correlation functions yields a new distribution of correlation times, we may investigate frames used to separate the total correlation different motions. We suggest several possible separations, and compare the results. In all cases, we remove local structural distortion (one-bond librations) by defining a frame that aligns the local structure. Then, we also test means of removing overall motion for the head group and backbone region (HG/BB), and also each chain. For the HG/BB, we try calculation of the longest component of the moment of inertia (MOI, see Supplementary Figure 9A) and RMS alignment of the backbone atoms (Supplementary Figure 9B). For the

chains, we separate the chains into upper and lower regions, calculating a MOI for each region (Supplementary Figure 9A), and also take the chains as a whole (Supplementary Figure 9B). We also attempt to separate internal motion into components. One approach is to use a C–C bond as frame (one of the C should be bonded to the corresponding H), thus separating rotation around the C–C bond from reorientation of the C–C bond (Supplementary Figure 9C). In a second approach, we separate motion parallel to the MOI from motion perpendicular to the MOI, by defining a frame that projects the bond direction onto the plane perpendicular to the MOI (Supplementary Figure 9D). The procedure for defining each frame is discussed below.

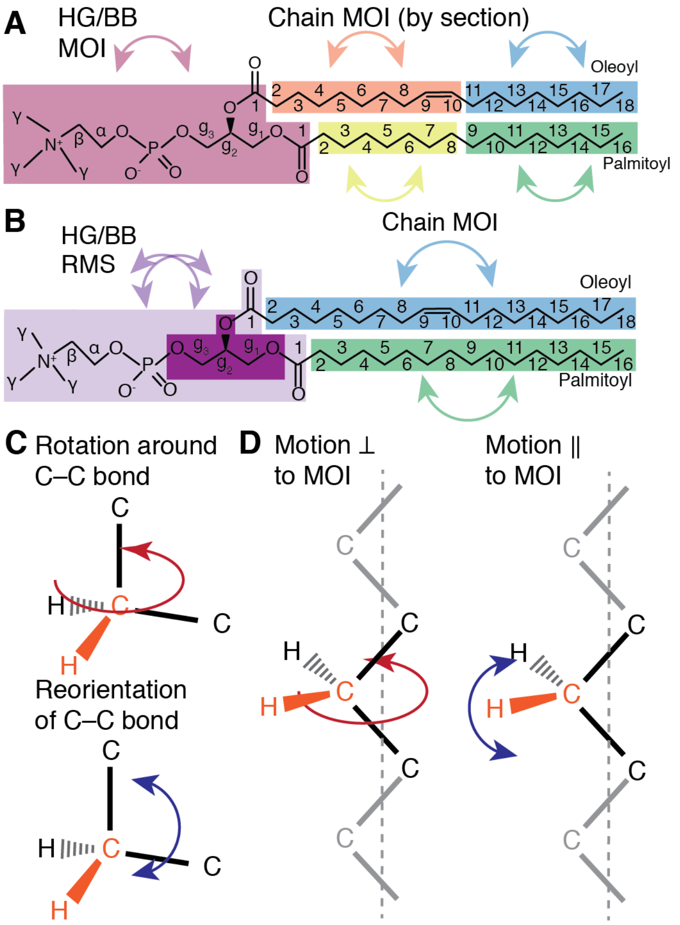

Supplementary Figure 9. Various definitions of frames. **A** illustrates frames defining overall motion, where POPC is separated into 5 groups: HG/BB, and chains each separated into an upper and lower part. For each group, the MOI is used to define overall motion. **B** illustrates another set of frames defining overall motion, where HG/BB motion is defined based on RMS alignment of the backbone atoms (g_1, g_2, g_3 and attached oxygen). Chains are taken as a whole instead of separating into parts. **C** illustrates separation of internal motion via alignment of the nearest C–C bond (separating rotation around the C–C bond from reorientation of the C–C bond). **D** illustrates separation of internal motions into components parallel and perpendicular to the MOI via the MOIxy frame (section 2.3)

2.1 Definition of the frames of NMR interactions

Defining the frame of the NMR interaction (dipolar coupling) requires defining a full axis system (*x*, *y*, and *z*-axes). While definition of the *z*-axis is usually obvious–for a one-bond dipole coupling, the *z*-axis should simply be along the bond–the direction of the *x*- and *y*-axes is less so. In fact, the choice is somewhat arbitrary, with the relatively simple requirement that the chosen axes move along with the NMR interaction. Then, for each H–C bond, we define the C–X (X being a heteronucleus, usually another C) leading towards the g_2 carbon to be in the *xz*-plane (for g_2, we take the g_1 carbon). Using the vector along *z* (the H–C) bond, and a vector in the *xz*-plane, it is straightforward to obtain the *x*- and *y*-axes (*y* is the cross-product of the *z* and *xz* vectors, *x* is the cross product of the *y* and *z* vectors). For the carbonyls, we assume the *z*-axis is along the C=O bond, and use the single bonded oxygen to define the *xz*-plane.

2.2 Definition of RMS frames

RMS frames (root-mean square alignment frames) are defined by the rotation required to rotate the current structure such that a selection of atoms are aligned to a reference structure (acquiring a linear-least squares fit). The librational frames and head group RMS frame use this approach. For the librational frames, for a given H–C bond (or C=O bond, for the carbonyl), we select the central carbon and all atoms bonded to it as our reference selection. The reference structure is simply taken as the positions of those atoms at the beginning of the trajectory. For the alignment of the head group, we select the three glycerol-backbone carbons (g_1, g_2, and g_3) and the three oxygen atoms bound to them.

To obtain vectors defining the frame at some time τ, we first subtract away the mean position of all atoms in the reference structure and in the atoms at time τ. We then solve for the rotation matrix that results in the best alignment of the reference atoms to the atoms at time τ (we perform this alignment to obtain the active rotation matrix from the reference positions to the current position). This matrix is obtained using the Kabsch algorithm.[15]

$$
\begin{aligned}
&v_{\mathrm{ref}}: \text{3xN matrix of the reference positions } (\mathrm{mean}(v_{\mathrm{ref}}) = [0,0,0]) \\
&v_{\tau}: \text{3xN matrix of positions at } \tau \ (\mathrm{mean}(v_{\tau}) = [0,0,0]) \\
&\mathbf{H} = v_{\mathrm{ref}} \cdot v_{\tau}^{\dagger}: \text{3x3 matrix}
\end{aligned}
\qquad (9)
$$

Once **H** is obtained, we acquire its singular value decomposition (SciPy linear algebra module[14]), from which we may calculate the rotation matrix.

$$
\begin{aligned}
&\mathbf{H} = \mathbf{U} \cdot \Sigma \cdot \mathbf{V}^* \\
&d = \mathrm{sign}\left(\det\left(V \cdot U^{\mathrm{T}}\right)\right) \\
&\mathbf{R}_{\mathrm{ZYZ}}(\Omega_{\mathrm{ref},\tau}) = V \cdot \begin{bmatrix} 1 & 0 & 0 \\ 0 & 1 & 0 \\ 0 & 0 & d \end{bmatrix} \cdot U^{\mathrm{T}}
\end{aligned}
\tag{10}
$$

Then, the frame is defined by vectors $v_{\mathrm{X}}(\tau), v_{\mathrm{Y}}(\tau)$, and $v_{\mathrm{Z}}(\tau)$ which are the columns of the rotation matrix.

2.3 Definition of MOI frames

In each of the two chains, we separate overall motion of the moment of inertia (MOI) from motion within the chain. Defining this frame is simply a matter of calculating the MOI of the chain (we only take the C nuclei), and extracting the largest component, which defines the *z*-axis of this frame. The MOI matrix (neglecting masses) is defined as

$$
\begin{aligned}
&I_{\alpha\beta} = \sum_{k=1}^{N} \left(\| \mathbf{r}_k \|^2 \delta_{ij} - r_{\alpha}^{(k)} r_{\beta}^{(k)} \right) \\
&\mathbf{r}_k = \left(r_{\mathrm{x}}^{(k)}, r_{\mathrm{y}}^{(k)}, r_{\mathrm{z}}^{(k)} \right)
\end{aligned}
\tag{11}
$$

,

where $\mathbf{r}_k$ is a vector to the point mass (center of mass). The direction of the largest component (*z*) may be obtained by computing the eigenvalues and eigenvectors of **I**, and then taking the vector corresponding to the largest eigenvalue.

2.4 Definition of bond frames

A bond frame is a frame defined simply by the direction of a bond. That is, we take the *z*-axis of the frame to be the bond direction. *x*- and *y*-axes are not required, and no further computation is necessary (aside from vector normalization).

2.5 Separating parallel and perpendicular motion: MOIxy frame

We define a frame to separate internal motion in the chains into components parallel and perpendicular to the MOI. This is achieved with a frame that projects the direction of the bond onto the plane that is perpendicular to the longest component of the MOI. If $\mathbf{v}_{\mathrm{Z}}(\tau)$ is the direction of the bond at time τ, and $v_{\mathrm{Z}}^{\mathrm{MOI}}(\tau)$ is the direction of the longest component of the MOI, then

$$
v_{\mathrm{Z}}^{\mathrm{MOIxy}}(\tau) = \mathbf{v}_{\mathrm{Z}}(\tau) - v_{\mathrm{Z}}^{\mathrm{MOI}}(\tau) * \left(\mathbf{v}_{\mathrm{Z}}(\tau) \cdot v_{\mathrm{Z}}^{\mathrm{MOI}}(\tau) \right). \tag{12}
$$

$\mathbf{v}_{Z}(\tau)\cdot v_{Z}^{\mathrm{MOI}}(\tau)$ is the dot product of the bond vector and the MOI (both should be normalized), such that $v_{Z}^{\mathrm{MOI}}(\tau)*\left(\mathbf{v}_{Z}(\tau)\cdot v_{Z}^{\mathrm{MOI}}(\tau)\right)$ is the projection of the bond vector onto the MOI. Then, taking the difference of the bond vector and this projection leaves only the component of the bond vector in the xy-plane behind. The resulting vector, $v_{Z}^{\mathrm{MOIxy}}(\tau)$, needs to be renormalized to one.

2.6 Frame screening

With our frame definitions, we may determine the best combination of frames for analyzing POPC motion. We calculate the correlation function for the total motion of each bond vector directly, and fit the correlation function using detector analysis, with the resulting detector responses shown in Supplementary Figure 10 (sensitivities in A, detector responses in B-E, where colored lines result from analysis of the total motion). Then, we calculate the product of correlation functions for separated motions, similarly fit with detector analysis (black lines in Supplementary Figure 10B-E) and compare to the directly calculated correlation function.

In Supplementary Figure 10B-E, we always include separation of one-bond librations. Then, in Supplementary Figure 10B,C, we try to separate overall motion from internal motion. In Supplementary Figure 10B, we use a MOI frame for the HG/BB, and also a MOI frame for the upper and lower halves of each chain (5 groups in total, see Supplementary Figure 9A). Agreement within the chains is fairly good, although backbone carbons exhibit severe disagreement with the MOI frame. Then, in Supplementary Figure 10C, we replace the MOI in the HG/BB with RMS alignment of the glycerol atoms (Supplementary Figure 9B). We also no longer separate the chains into upper and lower parts when applying the MOI frame. The changes yield significant improvement in the HG/BB, and minor improvements in the chains, so that we use RMS alignment of the glycerol in the HG/BB and take the MOI of each chain, without separation into upper and lower chains.

We continue to attempt to further separate internal motion into components. Our first attempt is to separate internal motion by taking a C–C bond (one C should be in the H–C bond), in order to separate rotation around the C–C bond from reorientation of the C–C bond (we always take the bond leading towards the g_2 position). The result is severe disagreement in Supplementary Figure 10D. This is the result of strong correlation between these two motions, and so we cannot use this frame separation. Finally, we separate motion within the two chains into components parallel and perpendicular to the MOI in Supplementary Figure 10E, adding only minor additional error compared to the directly

calculated correlation function. Note that carbonyls are included in the HG/BB grouping, and double bonded carbons are not split into parallel and perpendicular components (then, in the chains, we obtain 4 separated motions, but in the HG/BB, C’, and double bonded carbons, only 3 separated motions). We take this set of frames for separating the total motion into components.

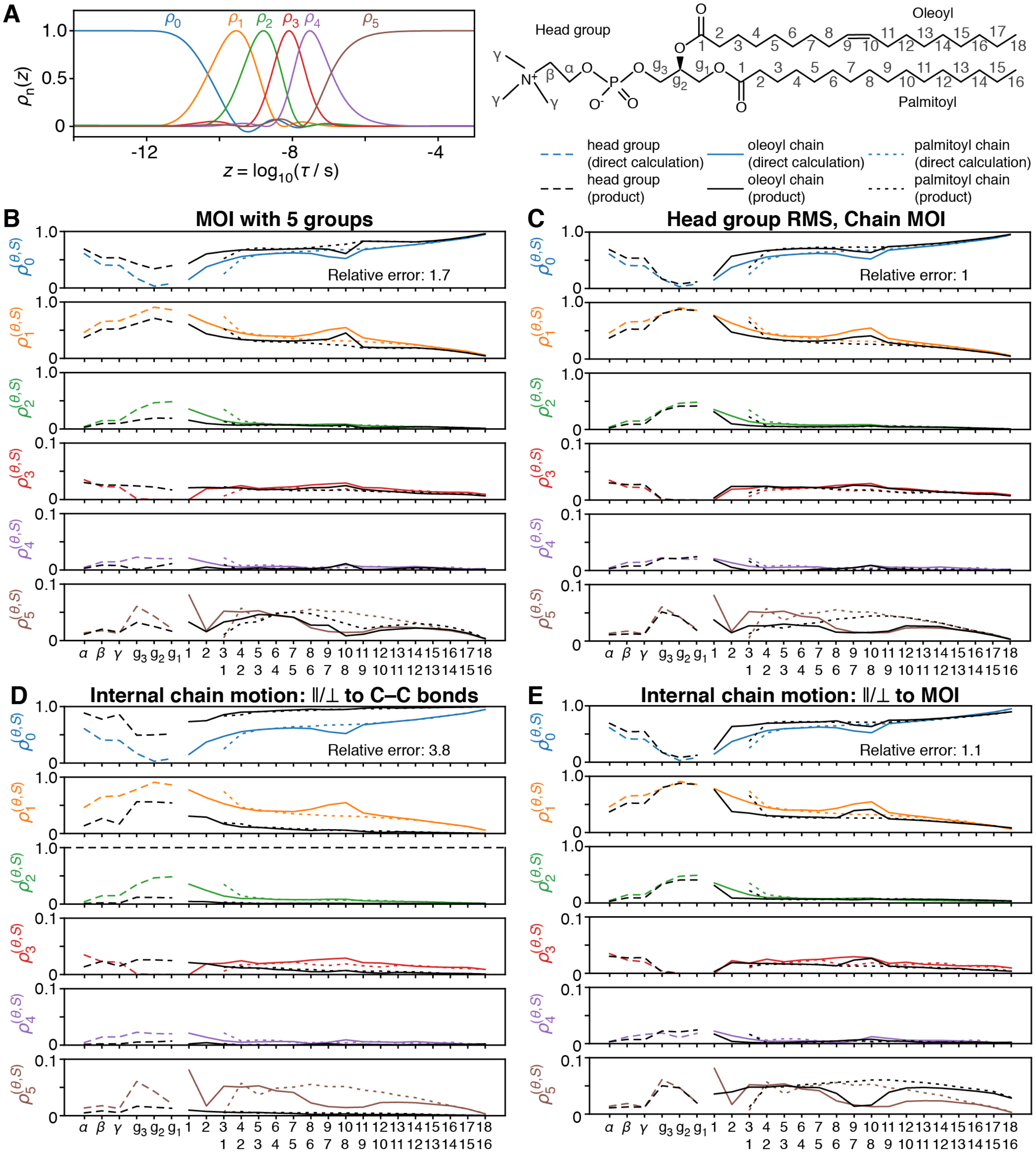

Supplementary Figure 10. Screening different combinations of frames. Frame screening is performed by calculating the total correlation function directly, and by taking a product of the correlation functions for separated motions. These are compared using detectors, where the corresponding sensitivities are found in **A**. Then, in **B-E**, a detector analysis of the total correlation function (color) is compared to a detector analysis of the product of correlation functions (black). HG/BB, oleoyl, and palmitoyl chains are indicated by dashed, solid, and dotted lines, respectively. Source data are provided as a Source Data File.

Supplementary Note 3: Movie descriptions

We use several short videos to help illustrate both detector responses and the behavior of residual tensors due to motions. We also plot the separation of motions using frames, juxtaposed both against residual tensors and against the resulting detector responses. Here we briefly describe how the various pieces of information are plotted in the videos.

3.1 Time indicator

In each video, a time indicator is displayed. This shows how much time is elapsed in the MD simulation within one second of the video. We log-space the MD frames shown in the video so that this value will change, allowing one to view different timescales of motion as the frame rate accelerates. For example, if the time indicator shows “1 s : 1 ns”, this means that from 0.5 s earlier (15 frames) in the video and 0.5 s later (15 frames), 1 ns of the MD trajectory will be covered. Then, each video covers 125 ns of trajectory, but the indicator will display a final value of 83 ns. Note that at the beginning of the video, we use “1 s : 2 ps”, but since we have saved frames only every 5 ps, resulting in some jumps at the beginning of the movies.

3.2 Plotting detector responses

In plots of detector responses (e.g. det_overall.mov), we see detector responses as 3D representations, with detector response encoded both as a color and atomic radius. The information displayed changes as the motion accelerates in the video, according to the following procedure:

1) We first take the correlation time corresponding to the current value on the time indicator, so that the motions taking about 1 s to occur are then highlighted with the detectors. We denote the log of this correlation time as z_c
2) We calculate the radius from the detector responses and z_c:

$$r = 0.9\ \text{Å} + (4\ \text{Å}) * \sum_{n=0}^{3} \rho_n(z_\mathrm{c}) \cdot \rho_n^{(\theta,S)} \qquad (13)$$

   Note that this is just a weighted sum of the detector responses. If we are at the maximum for detector *n*, then $r \approx 0.9\ \text{Å} + (4\ \text{Å})\rho_n^{(\theta,S)}$, since $\max(\rho_n(z)) = 1$. In practice, it will be slightly larger since the other detector sensitivities are not quite zero.
3) We calculate the color, first noting the colors assigned to each detector:

   ρ_0: Blue ($[C_{0r}, C_{0g}, C_{0b}] = [31,118,180]$)

   ρ_1: Orange ($[C_{1r}, C_{1g}, C_{1b}] = [255,127,14]$)

ρ_2: Green ($[C_{2r}, C_{2g}, C_{2b}] = [44,160,44]$)

ρ_3: Red ($[C_{1r}, C_{1g}, C_{1b}] = [214,39,40]$)

No response: Tan ($[C_{\mathrm{N}r}, C_{\mathrm{N}g}, C_{\mathrm{N}b}] = [210,180,140]$)

Then, the RGB value (on a scale 0-255) used is:

$$
\begin{aligned}
x_n &= \rho_n^{(\theta,S)} / \max(\rho_m^{(\theta,S)}) \\
R &= \left[1 - \sum_{n=0}^{3} \rho_n(z_\mathrm{c}) \cdot x_n\right] C_{\mathrm{N}r} + \sum_{n=0}^{3} \rho_n(z_\mathrm{c}) \cdot x_n C_{nr} \\
G &= \left[1 - \sum_{n=0}^{3} \rho_n(z_\mathrm{c}) \cdot x_n\right] C_{\mathrm{N}g} + \sum_{n=0}^{3} \rho_n(z_\mathrm{c}) \cdot x_n C_{ng} \\
B &= \left[1 - \sum_{n=0}^{3} \rho_n(z_\mathrm{c}) \cdot x_n\right] C_{\mathrm{N}b} + \sum_{n=0}^{3} \rho_n(z_\mathrm{c}) \cdot x_n C_{nb}
\end{aligned}
\tag{14}
$$

The normalized response, x_n, is obtained by dividing by the maximum of the detector responses for all bonds, and all detectors. Then, the RGB value is a weighted average of the four colors above, where the weighting depends on the size of the sensitivity of each detector at z_c and the detector response, and the color tan, corresponding to no response, where if the sum of detector responses times detector sensitivities is 1, then tan makes no contribution, but if the sum is zero, then the color is only tan. At the maximum of a given detector, for example ρ_2, the color is approximately a weighted average of green and tan, where if $\rho_2^{(\theta,S)} = 1$, then we will have just green, but if $\rho_2^{(\theta,S)} = 0$, then the color will be just tan.

3.3 Plotting tensors

Movies displaying tensors are simply showing the tensor magnitude (δ), shape (η), and orientation (α, β, γ) corresponding to a tensor averaged for a length of time corresponding to the current time indicator, and does not correspond to the amount of the trajectory elapsed. The length of the tensor in a given direction corresponds to the value of the z-component of the tensor if the molecule was rotated such that the given direction pointed along the z-axis. Positive values for the z-component are shown in red and negative values in blue.

Supplementary Note 4: Dynamic Landscape construction

Dynamic Landscapes of the individual motions are obtained by first calculating detector responses for each separated motion, shown in Supplementary Figure 12B, color. We use higher resolution detector sensitivities (Supplementary Figure 12A) than for direct comparison to experiment, to ensure that we obtain high quality fits of the MD-derived correlation functions. For each position, we then fit all 9 detector responses to a 3 parameter model, consisting of amplitude, $(1-S^2)$, correlation time of the maximum of the distribution, τ_{max}, and full-width at half-maximum (FWHM) of the distribution. For overall motions (Supplementary Figure 12E), we use a Gaussian distribution, otherwise we fit to a skewed Gaussian distribution (2x broader towards long correlation times). Note that detector analysis of correlation functions does not distinguish between very slow motion and no motion (i.e. S^2. Practically, $\rho_8^{(\theta,S)}$ is a good approximation for S^2, but strictly speaking, amplitude appearing in this detector may be from S^2 or from very slow motion). Therefore, we simply insert S^2 into the distribution at the longest correlation time, i.e. z=-3 ($\tau_c = 1\ \mathrm{ms}$). The resulting distributions, $\theta(z)$, therefore always integrate to 1.

Gaussian Distribution:

$$\sigma = \frac{\mathrm{FWHM}}{2\sqrt{2\ln 2}}$$

$$\theta(z) = \begin{cases} S^2 & z = -3 \\ (1-S^2)\dfrac{1}{\sigma\sqrt{2\pi}}\exp\left(-\dfrac{(z-z_{\mathrm{max}})^2}{2\sigma_{\mathrm{f}}^2}\right) & \text{otherwise} \end{cases}$$

Skewed Gaussian Distribution:

$$\sigma_{\mathrm{f}} = \frac{2}{3}\frac{\mathrm{FWHM}}{2\sqrt{2\ln 2}},\ \sigma_{\mathrm{s}} = \frac{4}{3}\frac{\mathrm{FWHM}}{2\sqrt{2\ln 2}},\ z_{\mathrm{max}} = \log_{10}(\tau_c\ /\ \mathrm{s}) \tag{15}$$

$$\theta(z) = \begin{cases} S^2 & z = -3 \\ (1-S^2)\dfrac{2}{3}\dfrac{1}{\sigma_{\mathrm{f}}\sqrt{2\pi}}\exp\left(-\dfrac{(z-z_{\mathrm{max}})^2}{2\sigma_{\mathrm{f}}^2}\right) & \text{if } z \le z_{\mathrm{max}} \\ (1-S^2)\dfrac{4}{3}\dfrac{1}{\sigma_{\mathrm{s}}\sqrt{2\pi}}\exp\left(-\dfrac{(z-z_{\mathrm{max}})^2}{2\sigma_{\mathrm{s}}^2}\right) & \text{otherwise} \end{cases}$$

4.1 Choice of functional form of the distribution

Nevzorov, Trouard, and Brown have shown that collective motions occurring over *d* dimensions results in a power law where the spectral density is proportional to[16]

$$J(\omega) \propto |\omega|^{-(2-d/2)}. \tag{16}$$

We note that this power law results from a distribution of correlation times having the following form

$$\begin{aligned} &\theta(z) \propto 10^{z(1-d/2)} \\ &d = 3 : \theta(z) \propto 10^{-z/2} \\ &d = 2 : \theta(z) \propto 1 \\ &d = 1 : \theta(z) \propto 10^{z/2} \end{aligned} \tag{17}$$

For d=3, increasing correlation times lead to a decreasing amplitude of the distribution, and conversely, for d=1, increasing correlation times lead to an increasing amplitude, whereas d=2 yields a uniform distribution. We may verify that $\theta(z) \propto 10^{z(1-d/2)}$ leads to a spectral density with the functional form in (21) by integrating the spectral density over the distribution

$$J(\omega) = \frac{2}{5}(1-S^2)\int_{-\infty}^{\infty} \theta(z)\frac{10^z \cdot \mathrm{s}}{1+(\omega \cdot 10^z \cdot \mathrm{s})^2}dz \tag{18}$$

The second term in the integral, $10^z \cdot \mathrm{s}/(1+(\omega \cdot 10^z \cdot \mathrm{s})^2)$, is symmetric around $z_0 = -\log_{10}(\omega)$, and so we assume $\theta(z)$ is approximately linear around this value (reasonable for a broad, monotonic distribution as expected for a power law), such that

$$\begin{aligned} &\theta(z) \propto 10^{z(1-d/2)} \approx 10^{z_0(1-d/2)} + m(z-z_0) = \omega^{-(1-d/2)} + m(z-z_0) \\ &z_0 = -\log_{10}(\omega) \\ &J(\omega) \propto \int_{-\infty}^{\infty}\left[\omega^{-(1-d/2)} + \underbrace{m(z-z_0)}_{\text{antisymmetric}}\right]\underbrace{\frac{10^z \cdot \mathrm{s}}{1+(\omega \cdot 10^z \cdot \mathrm{s})^2}}_{\text{symmetric}}dz \end{aligned} \tag{19}$$

The term $m(z-z_0)$ is antisymmetric about z_0, whereas $10^z \cdot \mathrm{s}/(1+(\omega \cdot 10^z \cdot \mathrm{s})^2)$ is symmetric about z_0, such that the integral of the product of these terms is zero. Then, the integral reduces to

$$
\begin{aligned}
J(\omega) &\propto \omega^{-(1-d/2)} \int_{-\infty}^{\infty} \frac{10^z \cdot \mathrm{s}}{1+(\omega \cdot 10^z \cdot \mathrm{s})^2} dz \\
x = \omega \cdot 10^z,\ & dx = \omega \cdot 10^z \log(10) dz \\
&= \frac{\omega^{-(2-d/2)}}{\log(10)} \underbrace{\int_0^{\infty} \frac{1}{1+x^2} dx}_{=\pi/2} \\
&= \frac{\omega^{-(2-d/2)}}{\log(10)} \frac{\pi}{2} \\
&\propto \omega^{-(2-d/2)}
\end{aligned}
\tag{20}
$$

A substitution of variables brings us to a familiar integral, and so we find that the result is proportional to $\omega^{-(2-d/2)}$, which is the power-law from Brown and coworkers.[16]

The original derivation by Brown and coworkers makes a few important approximations. First, the distribution of correlation times results from there being many modes of motion, each having a correlation time. However, one assumes these may span from zero to infinitely long wavelengths, whereas in reality, the modes have a minimum length (limited by the molecule size or bond lengths) and also a maximum length (limited at least by vesicle size). One consequence of this assumption is that the distributions themselves all integrate to infinity. A second assumption that may introduce some deviation of the real distribution from the ideal power law is the calculation of the amplitude of a given mode: the amplitude of a mode is given as $\langle |\delta \mathbf{n}(\mathbf{q},t)|^2 \rangle = kT / Kq^2$, as a result from the classical equipartition theorem ($\mathbf{q}$ is the wave vector, K the elastic constant).[16] Then, one assumes the resulting spectral density is thus proportional to the mode amplitude. However, this neglects that faster motions result in an effective interaction tensor that has been scaled, such that the influence of slower motions is less than would be estimated by the power law (the distribution should decay faster than predicted by the power law). For fast motion, this will have little effect, and if the total amplitude of motion is low (S^2 near to 1), the influence of such behavior will also be negligible, but in membranes, the total amplitude may be quite large. We should be clear: these assumptions should not significantly hinder the application of power-laws to experimental data, since one is typically interested in the behavior of R_1 or $R_{1\rho}$ at a relatively narrow range of correlation times. However, if we are to characterize the distribution over a wider range of correlation times, deviation of the integral of $\theta(z)$, in particular, can be problematic.

While a thorough investigation into the best functional form to describe the distribution of correlation times for collective motions is beyond the scope of this study, we note that for d=3, a skewed Gaussian distribution is a good estimate, as given in

Supplementary Equation (15). At short correlation times, we know the distribution should approach zero due to bounds on the minima mode length, although we do not have a particular functional form. As one approaches long correlation times, our function should decay slightly faster than predicted by the power law. In Supplementary Figure 11, we see that the skewed Gaussian distribution has the desired property, with particularly close correspondence to the power law for a full-width at half maximum of 2 (note that we later find this value to be close to the fitted widths for internal motion, see Supplementary Figure 13).

For *d*=2, we simply take an un-skewed Gaussian distribution. Clearly, this distribution cannot be actually uniform, since it would then integrate to infinity, but how it should decay to zero for short or long correlation times is not predicted by the power-law, so that we simply take a symmetric function.

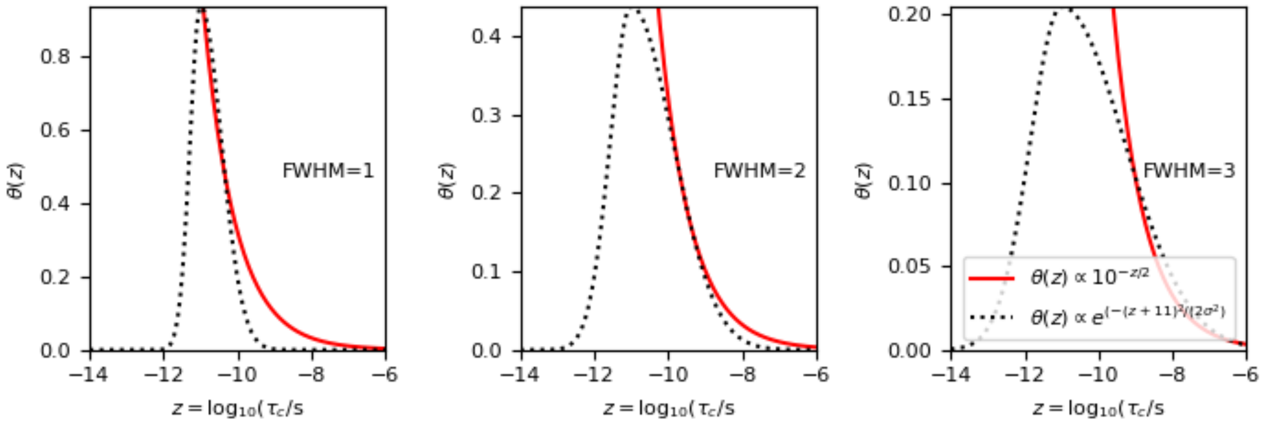

Supplementary Figure 11. Power law distribution vs. Gaussian distribution according to Supplementary Equation (15). In each plot, we show a skewed Gaussian distribution, with FWHM=1, 2, or 3 orders of magnitude, and compare this to the *d*=3 power law of $\theta(z)=10^{-z/2}$. Plots are normalized such that the two distributions intersect at the half-height of the Gaussian distribution.

4.2 Comparison of detector responses

Once the distribution is obtained, we may calculate the detector response, noting that S^2 has been absorbed into $\theta(z)$ in (15), and furthermore, the integral may be discretized.

$$\rho_n^{(\theta,S)}=\int_{-\infty}^{\infty}\theta(z)\rho_n(z)dz=\sum_i\theta(z_i)\rho_n(z_i)\Delta z \tag{21}$$

The fitted detector responses are shown in black in Supplementary Figure 12B, where we see very high quality agreement between detector responses for each separated motion and for their fitted values. The optimized parameters are shown in Supplementary Figure 13. Note that for librational motion, usually only $\rho_0^{(\theta,S)}$ and $\rho_8^{(\theta,S)}$ are non-zero. This means

that we know motion is very fast, but not precisely how fast. As a result, we have placed an upper bound on τ_{max} of 100 fs, and fixed the FWHM to 0.7 orders of magnitude.

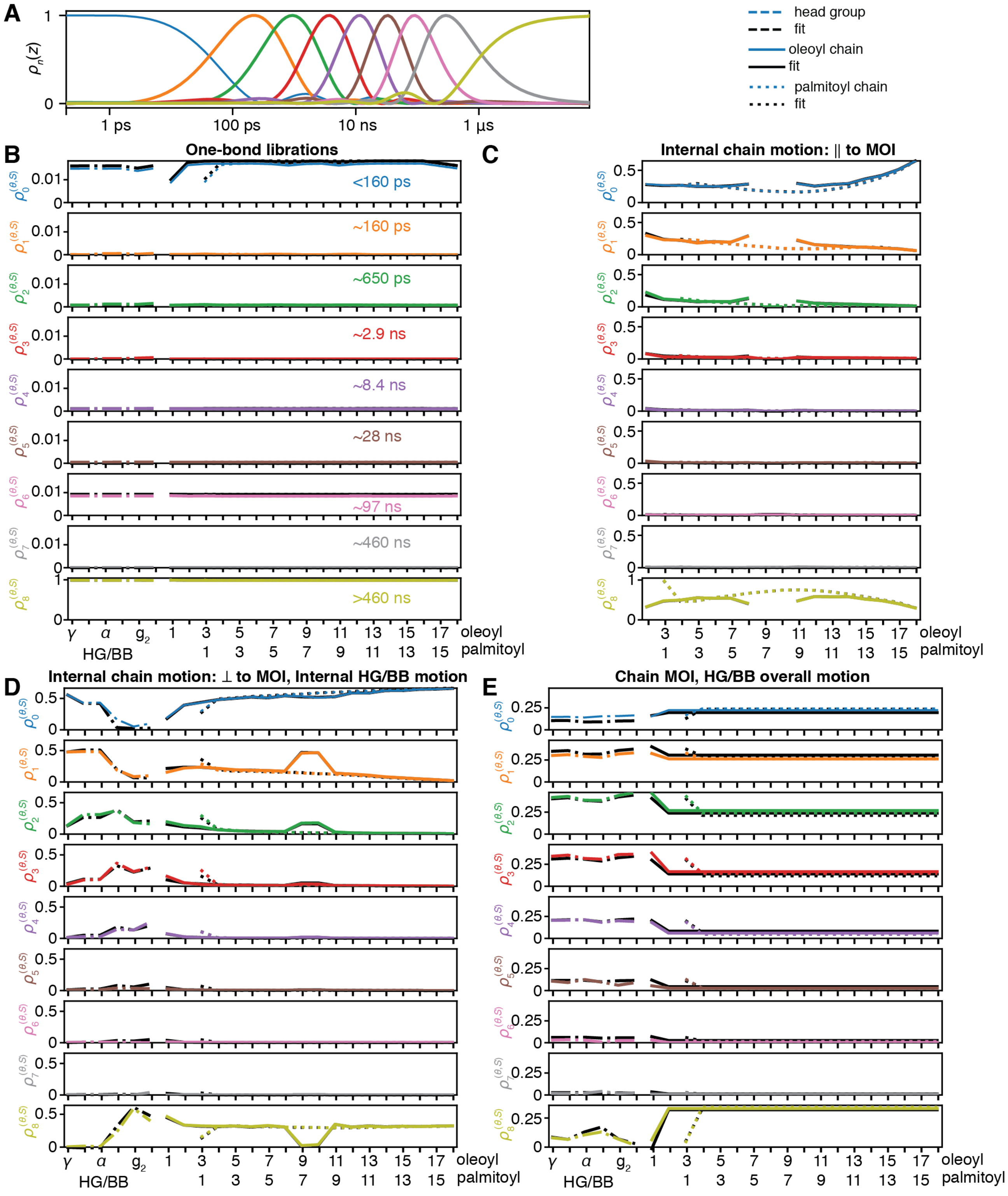

Supplementary Figure 12. Fits of detector responses for each motion to a simple, three-parameter distribution. To precisely describe motion within each frame, we use higher resolution detectors than have been applied in the main text for comparison to experiment, with the new detector sensitivities shown in **A**. Then, for each motion (**B**-**E**), we plot the resulting detector responses in color, and compare these to detector responses resulting from the fitted distributions (Supplementary Figure 13, Supplementary Figure 14). Source data are provided as a Source Data File.

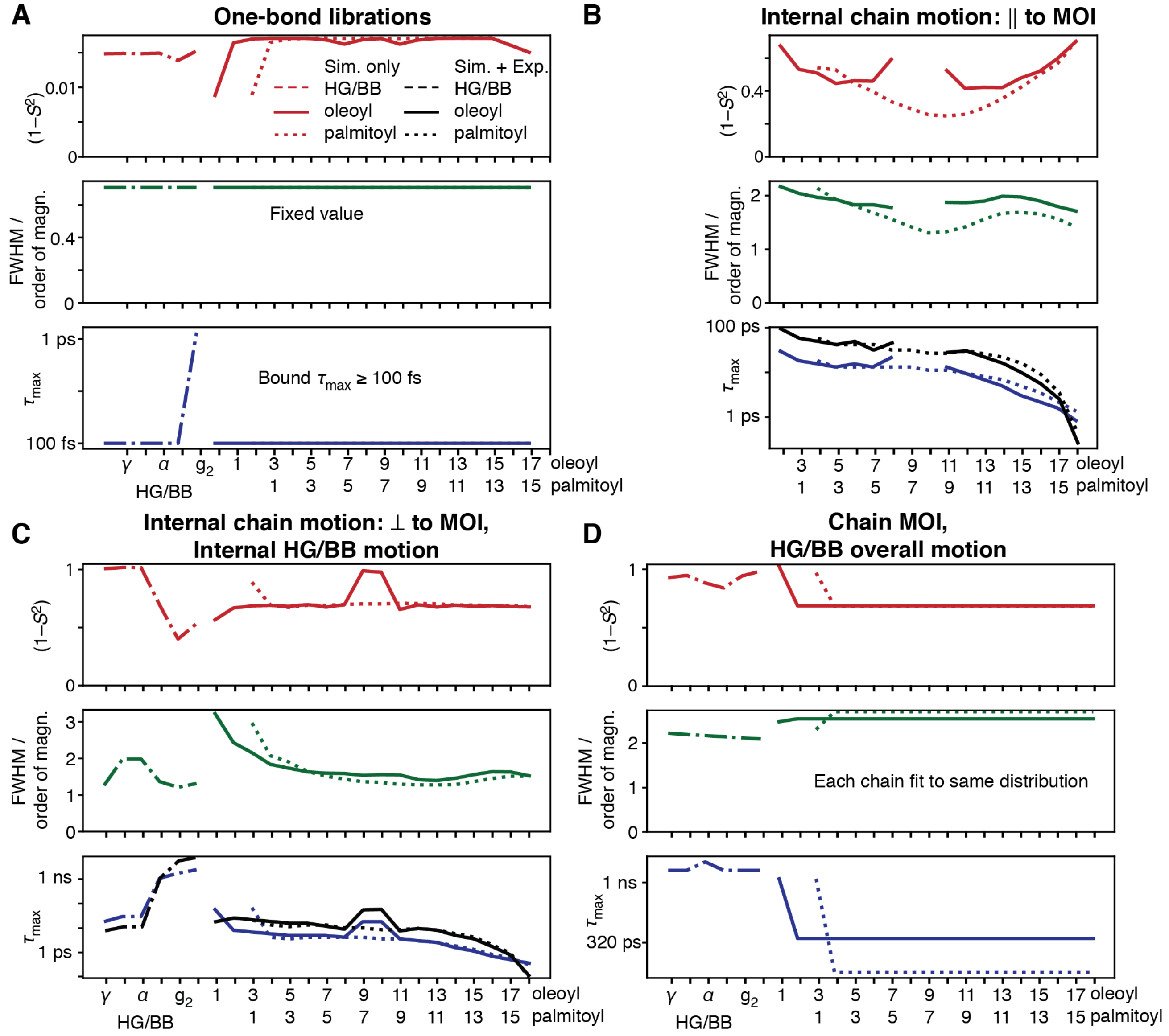

Supplementary Figure 13. Parameters for the dynamic landscapes. Colored lines show the MD-derived parameters for each type of motion in **A**-**D**. In **B** and **C**, parameters have been refined based on experimental detector response, where the new parameters are shown with black lines. Edited parameters **B** and **C** have been scaled by the same scaling factor. Additionally, positions for which experimental resonances overlap (see Supplementary Figure 1), the scaling factor is also constant for included resonances. Source data are provided as a Source Data File.

Then, the resulting landscape may be viewed for each motion, in Supplementary Figure 14A-D. For additional information, we plot the orientations sampled for selected bonds, in Supplementary Figure 14E. Note that inclusion of a distribution width is required for all except librational motion. In Supplementary Figure 15, we attempt to fit the detector responses only with an amplitude and single correlation time, where we find severe disagreement always in at least one detector. For internal chain motion parallel to the MOI (Supplementary Figure 15C), this disagreement is found for ρ_2. For motion perpendicular to the MOI and internal HG/BB motion, disagreement is found for ρ_2 and also in ρ_9 for the HG/BB. For overall motion, HG/BB disagreement is poor throughout, although is worst at ρ_4 and ρ_9, and within the chains, agreement is worst for ρ_2 and ρ_9.

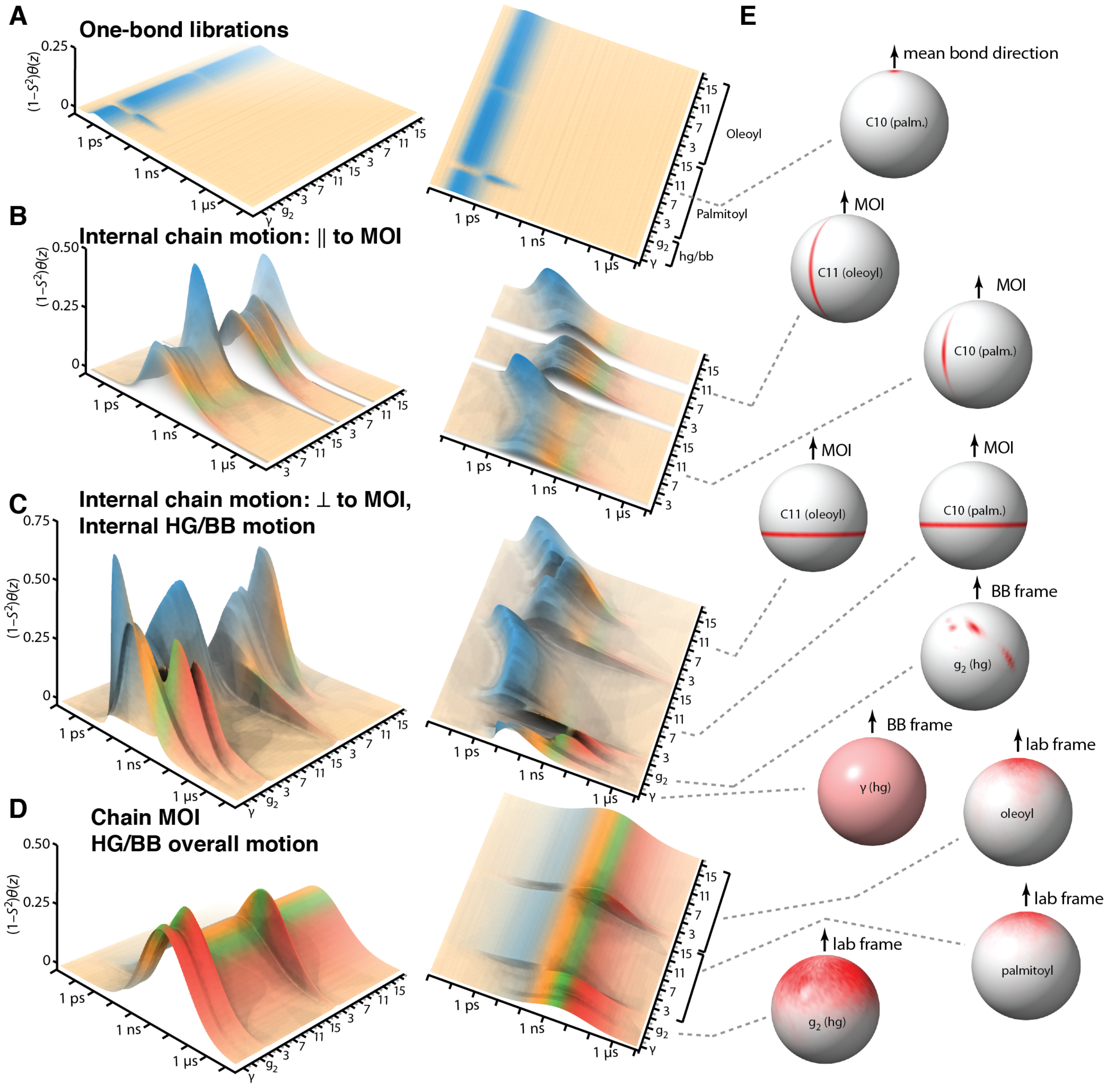

Supplementary Figure 14. Components of the MD-derived dynamic landscape. **A-D** plot fitted distributions of motions for each of 3-4 motions. Axes are the correlation time (left), and position, ordered from head group, backbone, palmitoyl chain, and oleoyl chain. The coloring corresponds to the detector most sensitive to a given correlation time (blue: ρ_0, orange: ρ_1, green: ρ_2, red: ρ_3), where the intensity of the color is determined by the amplitude at the corresponding correlation time (fades to tan for small amplitudes). **E** plots histograms of the orientations sampled for selected bonds for each of the motions. Histogram is mapped onto a sphere, where vertical arrows and names indicate what frame the orientations are given (either the frame of the mean bond direction for librations, the MOI or BB frames for internal motions, and the lab frame for overall, i.e. HG/BB and MOI motion). Note that all residual tensors in the chains point along the chain MOI, so that sampling of orientations due to MOI motion is identical for all bonds. In contrast, internal HG/BB motion does not result in tensors pointing in a uniform direction, so sampling due to the overall HG/BB motion can be different for different bonds.

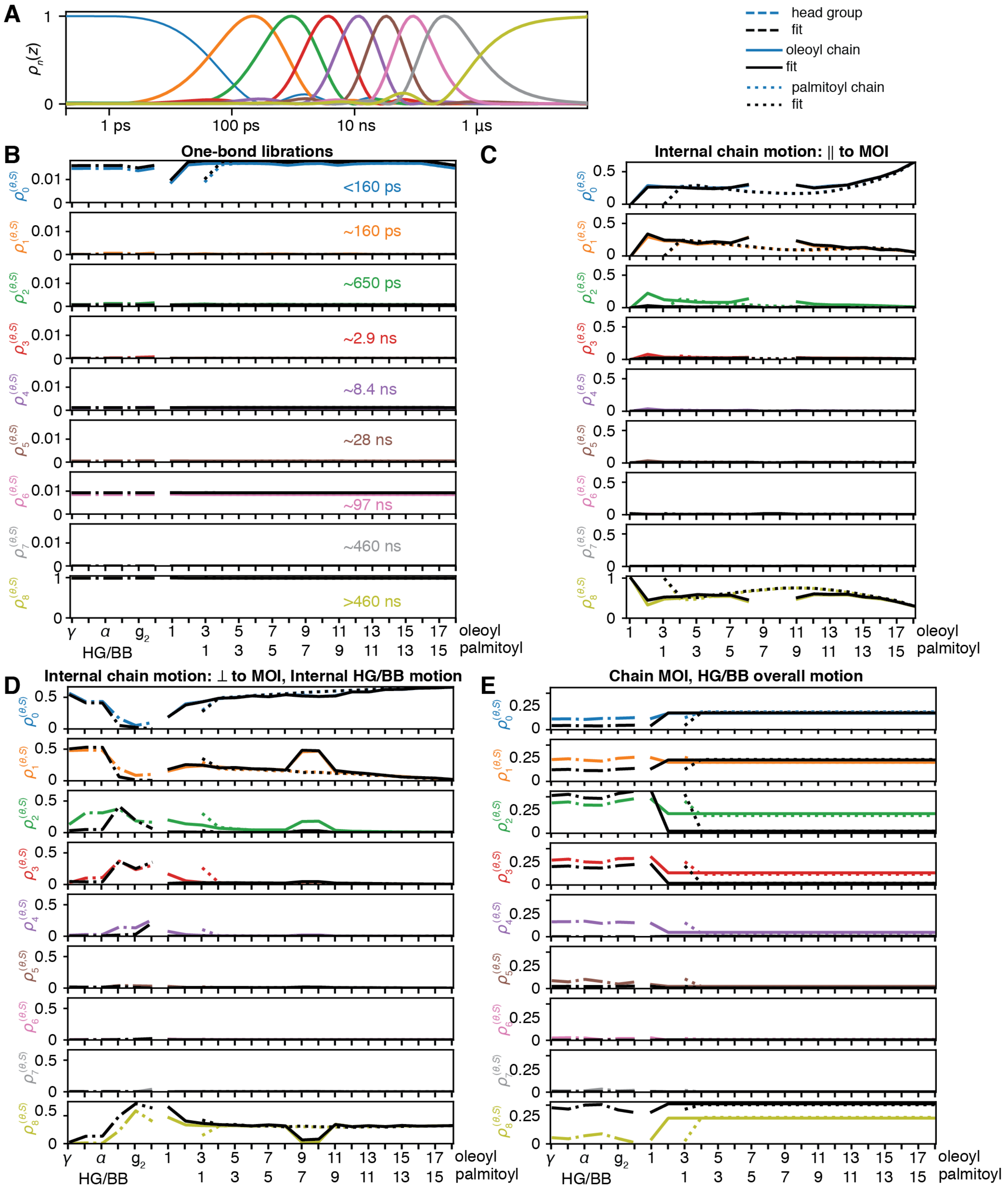

Supplementary Figure 15. Fits of detector responses for each motion to a two-parameter distribution (FWHM=0). Detector sensitivities shown are in **A**. Then, for each motion (**B**-**E**), we plot the resulting detector responses in color, and compare these to detector responses resulting from the fitted distributions, which only include and amplitude and correlation time, but no width. Results should be compared to Supplementary Figure 12. Source data are provided as a Source Data File.

Having fitted all detector responses resulting from the individual motions, using only three parameters for each motion, we now calculate the total distribution of motion. The total motion is the product of the individual correlation functions, such that

$$
\begin{aligned}
C(t) &= C_1(t)\cdot C_2(t)\cdot\ldots \\
&= \prod_n S_n^2 + (1-S_n^2)\int_{-\infty}^{\infty}\theta_n(z)\exp(-t/(10^z\cdot 1\,\mathrm{s}))dz \\
&= \left[S_1^2+(1-S_1^2)\sum_i A_{1,i}\exp(-t/(10^{z_i}\cdot 1\,\mathrm{s}))\right]\cdot\left[S_2^2+(1-S_2^2)\sum_i A_{1,i}\exp(-t/(10^{z_i}\cdot 1\,\mathrm{s}))\right]\cdot\ldots \\
&= \left[S_1^2S_2^2+S_2^2(1-S_1^2)\sum_i A_{1,i}\exp(-t/(10^{z_i}\cdot 1\,\mathrm{s}))+S_1^2(1-S_2^2)\sum_i A_{2,i}\exp(-t/(10^{z_i}\cdot 1\,\mathrm{s}))\right. \\
&\qquad\left.+(1-S_1^2)(1-S_2^2)\sum_i\sum_j A_{1,i}A_{2,i}\exp(-t/(10^{z_{i,j}^{\mathrm{eff}}}\cdot 1\,\mathrm{s}))\right]\cdot\ldots \\
z_{i,j}^{\mathrm{eff}} &= \log_{10}\left(\frac{1}{1/10^{z_i}+1/10^{z_j}}\right) = -\log_{10}\left(\frac{10^{z_i}+10^{z_j}}{10^{z_i+z_j}}\right) \\
&= z_i + z_j - \log_{10}(10^{z_i}+10^{z_j})
\end{aligned}
\tag{22}
$$

For each correlation function, we insert the discretized form of the integral over the distribution of motion, and multiply through for the first two correlation functions. The results of the three summations may be re-binned numerically into a new distribution that results from the product of the two correlation functions. This process is repeated until we have the distribution corresponding to the product of all individual motions. The result is shown in Supplementary Figure 16.

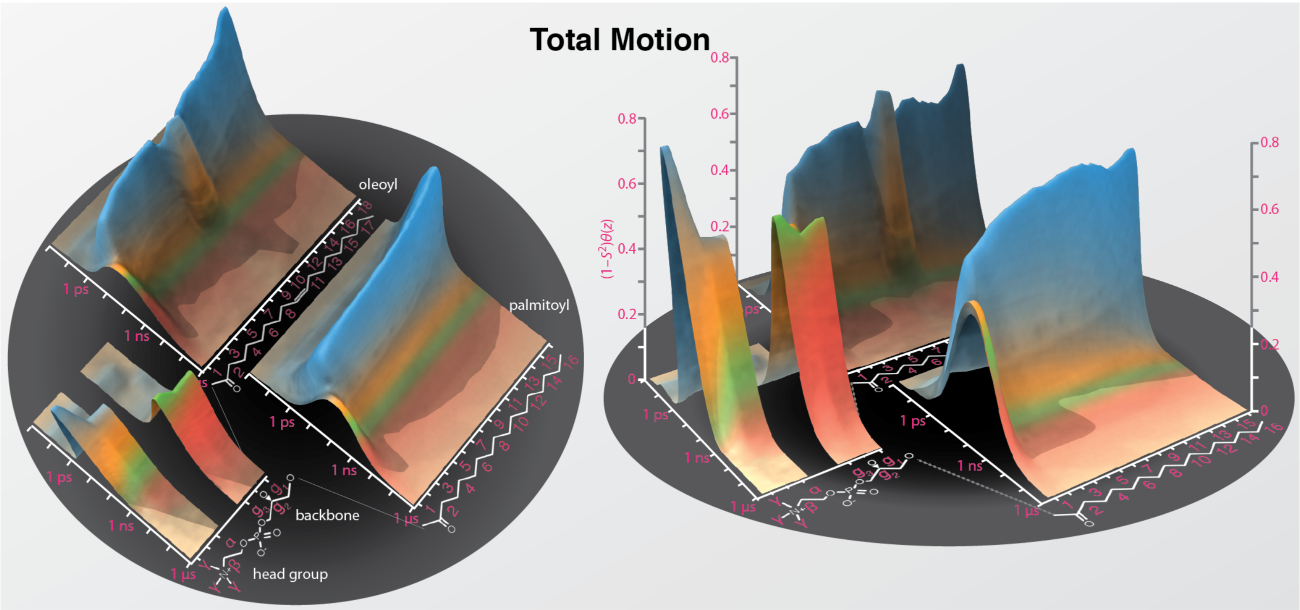

Supplementary Figure 16. Dynamic landscape of POPC membranes using MD only. Here we plot the product (Supplementary Equation (22)) of distributions found in Supplementary Figure 14. The plot is broken into components corresponding to the head group, backbone, oleoyl, and palmitoyl chains, with two perspectives of the landscape shown. Source data are provided as a Source Data File.

Now that we have the total distribution of motion, it is finally possible to calculate detector responses resulting from that distribution, using Supplementary Equation (21), and

compared to the experimental detector responses. Note that we average MD results over positions that have overlapping resonances experimentally. The results are shown in Supplementary Figure 17, with experimental results in color, and responses from the MD-derived landscape shown as grey, dashed lines.

While the results are very good, considering the multiple layers of complexity in this analysis, we would like to finally refine the dynamic landscape using the NMR experimental data. We cannot simply vary all parameters of the landscape because the number of free parameters would outnumber the amount of experimental data obtained. On the other hand, it is reasonable to vary a single free parameter for each measured resonance. In Supplementary Figure 17, we see the largest disagreement for $\rho_0^{(\theta,S)}$ and $\rho_1^{(\theta,S)}$, suggesting that we should adjust the internal motions, since these have the largest detector responses for ρ_0 and ρ_1. Furthermore, we find that simulation usually overestimates $\rho_0^{(\theta,S)}$ and underestimates $\rho_1^{(\theta,S)}$, which points to a disagreement in correlation time. Therefore, at each position, we vary τ_{max} for the internal motion. Within chains, where motion is separated into parallel and perpendicular components, we will vary the correlation time of both motions using the same scaling factor, and for positions having experimentally overlapping resonances, we will vary all correlation times also by a single scaling factor. Therefore, we still only have a single free parameter per resonance.

The results are shown in Supplementary Figure 17, as a black, dotted line. We obtain significant improvement in agreement by varying only the correlation time. The corrected landscapes for the individual motions and the product of all internal motions are shown in the main text, Fig. 5.

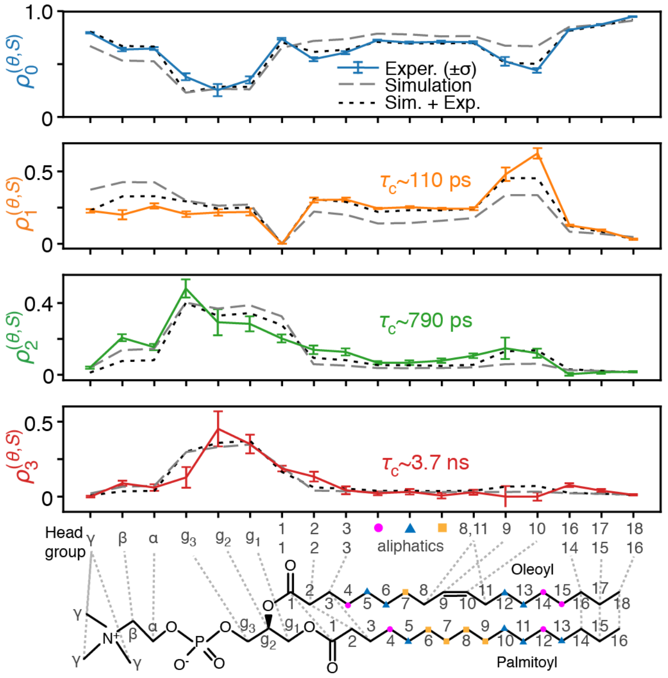

Supplementary Figure 17. Experimental detector responses (color) vs. responses derived from the dynamic landscape in Supplementary Figure 16 (MD data only, grey, dashed lines), and responses derived from the experimentally refined landscapes in main text Fig. 5 (black, dotted lines). Error bars represent $\pm\sigma$ of the experimental detector response, obtained via linear propagation-of-error from error determined for experimental rate constants (see Supplementary Note 1.3 for experimental error determination). Source data are provided as a Source Data File.

Supplementary Note 5: Further analysis

5.1 Configurational dynamics of the glycerol backbone

In Supplementary Figure 18, we plot the average free energy as a function of the β and γ Euler angles for internal motion of one H–C bond of the g_1, g_2, and g_3 positions. The relative free energy is calculated based on the relative population for each set of angles (over $1\text{x}10^5$ frames of the trajectory). Note that where the population is zero, strictly speaking, we cannot estimate the free energy, only that it is significantly higher than elsewhere. Then, we see 6 distinct energy minima traversed by the glycerol backbone. The relative populations of these states, and the angles sampled determine $|S|$ for the internal motion of g_1, g_2, and g_3.

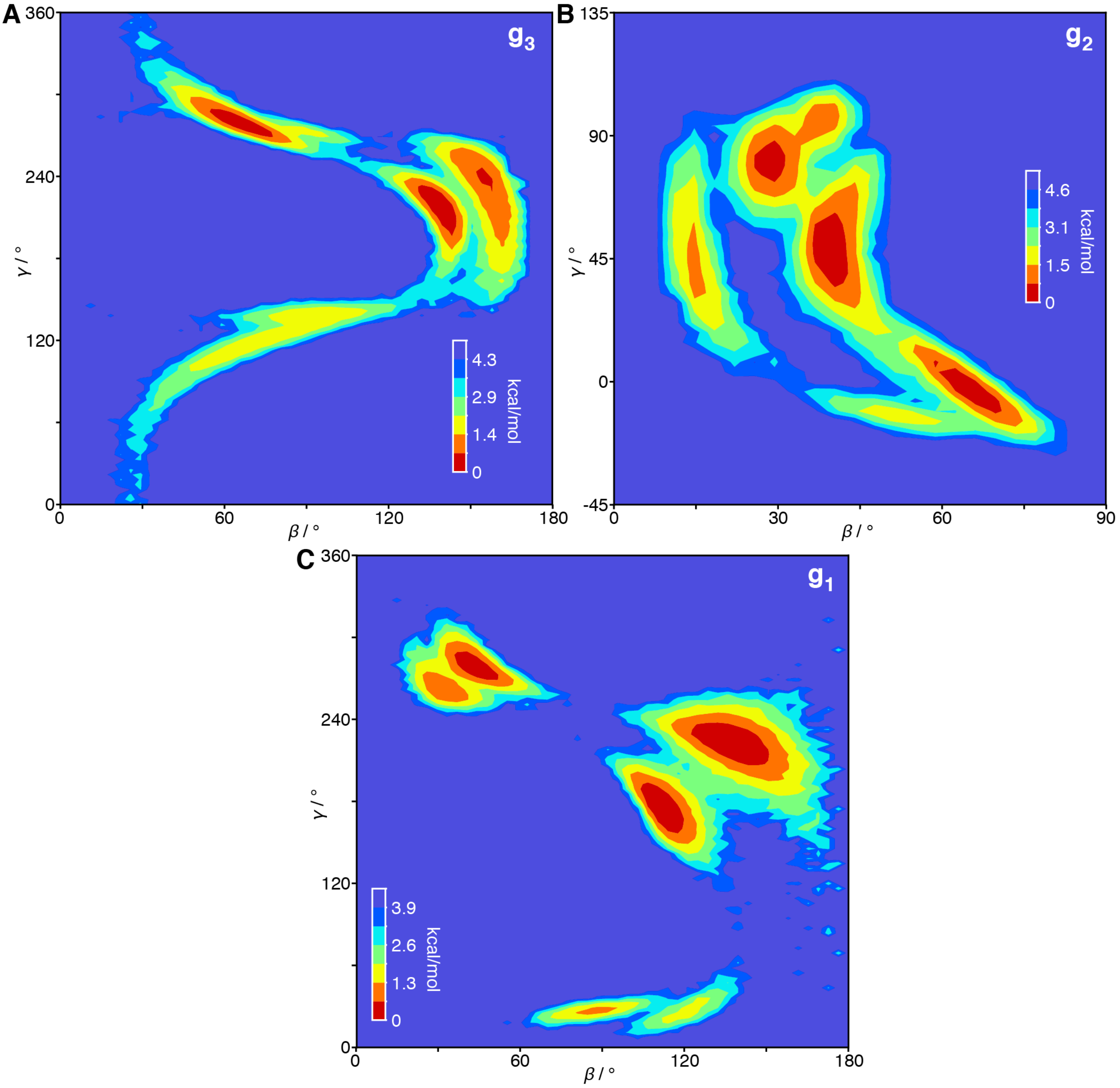

Supplementary Figure 18. Relative free energy vs. β/γ angles for glycerol backbone. **A-C** plots the relative free energy for each orientation of the β and γ angles. Most angles are unpopulated, corresponding to a high, albeit not precisely determined free energy. Other energies are shown via coloring (legend in each plot).

5.2 S^2 vs. $1/T_1$ via detectors

If we suppose that T_1 relaxation depends on some correlation function, such that

$$\begin{aligned} C(t) &= C_\mathrm{f}(t)C_\mathrm{s}(t) \\ &= \left[S_\mathrm{f}^2 + (1-S_\mathrm{f}^2)\sum_i A_i e^{-t/\tau_i^\mathrm{f}}\right]\left[S_\mathrm{s}^2 + (1-S_\mathrm{s}^2)\sum_j A_j e^{-t/\tau_j^\mathrm{s}}\right] \end{aligned} \tag{23}$$

If all fast motion is too fast to induce significant T_1 relaxation, we may then approximate the resulting spectral density as follows:

$$J(\omega) = S_f^2 \underbrace{(1 - S_s^2)\frac{2}{5}\sum_j A_j \frac{\tau_j^s}{1 + (\omega\tau_j^s)^2}}_{J_s(\omega)} \tag{24}$$

We have labeled the latter term $J_s(\omega)$, and if, for ω sampled by the given T_1 experiment, this function is relatively uniform in the chains, then we find that $1/T_1$ is proportional to S_f^2. If, furthermore, most motion is fast, then $S_f^2 \approx S^2$. Since $\rho_0^{(\theta,S)}$ is optimized to sample only fast motions (experimentally, we may also sample some slow motion, but these are relatively low amplitude), it is a better estimator of S_f^2 than S^2. Then, we can observe the relationship between $1-\rho_0^{(\theta,S)}$ and $\rho_1^{(\theta,S)}$, using either experimental data or detector responses derived from the dynamic landscape in text Fig. 6E. The result is shown in Supplementary Figure 19, where the linear relationship is well maintained both for experimentally derived detectors and those obtained via the dynamic landscape.

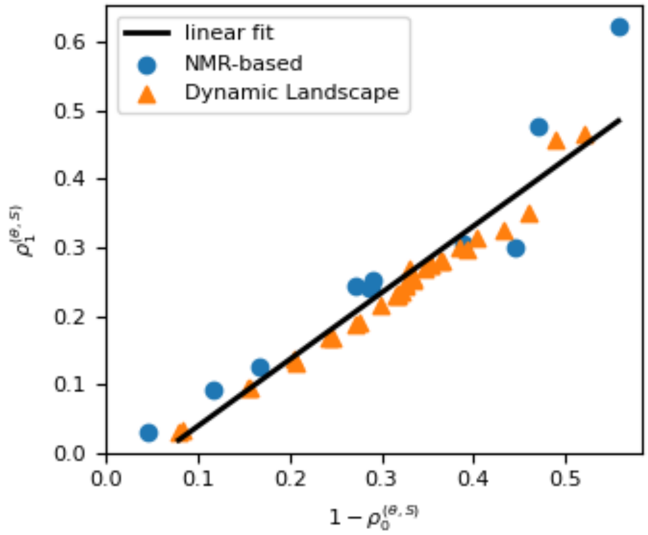

Supplementary Figure 19. $\rho_1^{(\theta,S)}$ vs. $1-\rho_0^{(\theta,S)}$. We plot $\rho_1^{(\theta,S)}$ vs. $1-\rho_0^{(\theta,S)}$ for all carbons in the chains (excluding carbonyls), where detectors were derived experimentally (blue circles) or from the dynamic landscape in text Fig. 6E (orange triangles). A black line indicates a linear fit of all data. Source data are provided as a Source Data File.

Supplementary References

1. Seelig, J. & Waespe-Sarcevic, N. Molecular order in cis and trans unsaturated phospholipid bilayers. *Biochemistry* **17**, 3310–3315 (1978).
2. Leftin, A., Molugu, T. R., Job, C., Beyer, K. & Brown, M. F. Area per Lipid and Cholesterol Interactions in Membranes from Separated Local-Field 13C NMR Spectroscopy. *Biophysical Journal* **107**, 2274–2286 (2014).
3. Ferreira, T. M. *et al.* Cholesterol and POPC segmental order parameters in lipid membranes: solid state 1H-13C NMR and MD simulation studies. *Phys Chem Chem Phys* vol. 15 1976–89 (2013).
4. Smith, A. A. INFOS: spectrum fitting software for NMR analysis. *J. Biomol. NMR* **67**, 77–94 (2017).

5. Kurbanov, R., Zinkevich, T. & Krushelnitsky, A. The nuclear magnetic resonance relaxation data analysis in solids: General R 1/R 1 equations and the model-free approach. *J. Chem. Phys.* vol. 135 184104 (1–9) (2011).
6. Munowitz, M. G., Griffin, R. G., Bodenhausen, G. & Huang, T. H. Two-dimensional rotational spin-echo nuclear magnetic resonance in solids: correlation of chemical shift and dipolar interactions. *J. Am. Chem. Soc.* **103**, 2529–2533 (1981).
7. Bielecki, A., Kolbert, A. C., De groot, H. J. M., Griffin, R. G. & Levitt, M. H. Frequency-Switched Lee—Goldburg Sequences in Solids. in *Advances in Magnetic and Optical Resonance* (ed. Warren, W. S.) vol. 14 111–124 (Academic Press, 1990).
8. Efron, B. Bootstrap Methods: Another Look at the Jackknife. *The Annals of Statistics* vol. 7 1–26 (1979).
9. Smith, A. A., Ernst, M. & Meier, B. H. Optimized 'detectors' for dynamics analysis in solid-state NMR. *J. Chem. Phys.* vol. 148 045104 (2018).
10. Smith, A. A., Ernst, M., Meier, B. H. & Ferrage, F. Reducing bias in the analysis of solution-state NMR data with dynamics detectors. *J. Chem. Phys.* vol. 151 034102 (2019).
11. Smith, A. A. How wide is the window opened by high-resolution relaxometry on the internal dynamics of proteins in solution? *J. Biomol. NMR* **75**, 119–131 (2021).
12. Smith, A. A., Ernst, M., Riniker, S. & Meier, B. H. Localized and collective motions in HET-s(218-289) fibrils from combined NMR relaxation and MD simulation. *Angew. Chem. Int. Ed.* vol. 58 9483–9488 (2019).
13. Harris, C. R. Array programming with NumPy. 6.
14. Virtanen, P. SciPy 1.0: fundamental algorithms for scientific computing in Python. *Nat. Methods* vol. 17 261–272 (2020).
15. Kabsch, W. A solution for the best rotation to relate two sets of vectors. *Acta. Cryst.* vol. A32 922–923 (1976).
16. Nevzorov, A. A., Trouard, T. P. & Brown, M. Correlation functions for lipid membrane dynamics obtained from NMR spectroscopy. *Phys. Rev. E* vol. 55 3276–3282 (1997).
