# Supplementary material for: A method to construct the dynamic landscape of a bio-membrane with experiment and simulation
Source: Nat Commun. 2022 Jan 10;13:108. doi: 10.1038/s41467-021-27417-y (PMC8748619; doi:10.1038/s41467-021-27417-y)
Supplement: Supplementary file 3 — Description of Additional Supplementary Files [file 41467_2021_27417_MOESM3_ESM.docx]

File Name: Source Data.zip

Description:

A collection of tab-separated text files contained plotted data from Figures 1, 2, 4, 5, and 6E, as well as Supplementary Figures 5, 7B, 8, 10, 12, 13, 15, 16, 17, and 19. Each text file has a short description at the top of the file as well as sub-headings in case of multi-part figures.

File Name: Supplementary Information

Description:

Additional figures, tables, and details of analysis.

File Name: Supplementary Movie 1

Description:

Detectors for the total motion. Detector responses are encoded (via color and radius) onto a POPC molecule that is extracted from the MD trajectory (left). The timescale indicator (bottom) shows how much of the trajectory is currently traversed in 1s of the movie. Color intensity and atom radii indicate the magnitude of the detector responses, where the detector responses shown at a given time are those corresponding to the correlation time shown in the timescale indicator. Where the correlation time falls between detectors, the responses and colors are averaged, with details in Supplementary Note 3.2. The detector sensitivities and responses are also plotted (right), where the current correlation time is indicated on the sensitivities (top, right), and responses are scaled (color) according to the current timescale (unscaled responses shown in grey).

File Name: Supplementary Movie 2

Description:

Detectors for librational motion. Detector responses resulting from one-bond librational motion are plotted onto a POPC molecule extracted from the MD trajectory. Plotting is the same as for "Detectors for the total motion" (Supplementary Movie 1), although we have modified the trajectory to only show the librational motion.

File Name: Supplementary Movie 3

Description:

Detectors for motion parallel to the MOI. Detector responses resulting from motion in the chains parallel to the MOI are plotted onto a POPC molecule extracted from the MD trajectory. Plotting is the same as for "Detectors for the total motion" (Supplementary Movie 1), although we have modified the trajectory to only show the motion parallel to the MOI. Note that this separation causes structures of the POPC molecule that are not physically possible (i.e. we cannot really have motion perpendicular to the MOI without having motion parallel to the MOI).

File Name: Supplementary Movie 4

Description:

Detectors for motion perpendicular to the MOI and internal motion of the HG/BB. Detector responses resulting from motion in the chains perpendicular to the MOI and from internal motion in the HG/BB are plotted onto a POPC molecule extracted from the MD trajectory. Plotting is the same as for "Detectors for the total motion" (Supplementary Movie 1), although we have modified the trajectory to only show the motion perpendicular to the MOI or internal motion in the HG/BB. Note that this separation causes structures of the POPC molecule that are not physically possible (i.e. we cannot really have motion perpendicular to the MOI without having motion parallel to the MOI).

File Name: Supplementary Movie 5

Description:

Detectors for overall motion of the chains and HG/BB. Detector responses resulting from motion overall motion of the chains (MOI) and the HG/BB (RMS). Plotting is the same as for "Detectors for the total motion" (Supplementary Movie 1), although we have modified the trajectory to only show the overall motions of each region of the molecule.

File Name: Supplementary Movie 6

Description:

Detectors for all motions. We re-plot the POPC molecules and detector responses from each frame, along with the total motion (from left to right: librations, motion parallel to the MOI, motion perpendicular to the MOI and internal HG/BB motion, overall motion of the chains (MOI) and overall motion of the HG/BB, total motion. This video allows one to see how the total motion is the product of all individual motions, and how the total detector responses result from the detector responses of the individual motions.

File Name: Supplementary Movie 7

Description:

Tensors for the total motion. Time-dependent residual tensors are shown for the total motion. Tensors are plotted in the middle of the corresponding bond, where color indicates the sign of the tensor at a given orientation (red: positive, blue: negative).

File Name: Supplementary Movie 8

Description:

Tensors for librational motion. Time-dependent residual tensors are shown for the total motion. Tensors are plotted in the middle of the corresponding bond, where color indicates the sign of the tensor at a given orientation (red: positive, blue: negative). We have modified the trajectory to only show the librational motion.

File Name: Supplementary Movie 9

Description:

Tensors for motion parallel to the MOI. Time-dependent residual tensors are shown for motion in the chains parallel to the MOI. Tensors are plotted in the middle of the corresponding bond, where color indicates the sign of the tensor at a given orientation (red: positive, blue: negative). We have modified the trajectory to only show the motion parallel to the MOI.

File Name: Supplementary Movie 10

Description:

Tensors for motion perpendicular to the MOI and internal motion of the HG/BB. Time-dependent residual tensors are shown for motion in the chains perpendicular to the MOI and for internal motion in the HG/BB. Tensors are plotted in the middle of the corresponding bond, where color indicates the sign of the tensor at a given orientation (red: positive, blue: negative). We have modified the trajectory to only show the corresponding motions.

File Name: Supplementary Movie 11

Description:

Tensors for the overall motion of the chains and HG/BB. Time-dependent residual tensors are shown for overall motions of the chains (MOI) and the HG/BB (RMS). Tensors are plotted in the middle of the corresponding bond, where color indicates the sign of the tensor at a given orientation (red: positive, blue: negative). We have modified the trajectory to only show the overall motions of each region of the molecule.
