# Supplementary material for: A method to construct the dynamic landscape of a bio-membrane with experiment and simulation
Source: Nat Commun. 2022 Jan 10;13:108. doi: 10.1038/s41467-021-27417-y (PMC8748619; doi:10.1038/s41467-021-27417-y)
Supplement: Supplementary file 15 — Reporting Summary [file 41467_2021_27417_MOESM15_ESM.pdf]

nature portfolio

Corresponding author(s): Albert A. Smith

Last updated by author(s): Nov 5, 2021

Reporting Summary

Nature Portfolio wishes to improve the reproducibility of the work that we publish. This form provides structure for consistency and transparency in reporting. For further information on Nature Portfolio policies, see our Editorial Policies and the Editorial Policy Checklist.

Statistics

For all statistical analyses, confirm that the following items are present in the figure legend, table legend, main text, or Methods section.

n/a	Confirmed	
☐	☒	The exact sample size (*n*) for each experimental group/condition, given as a discrete number and unit of measurement
☒	☐	A statement on whether measurements were taken from distinct samples or whether the same sample was measured repeatedly
☒	☐	The statistical test(s) used AND whether they are one- or two-sided<br>*Only common tests should be described solely by name; describe more complex techniques in the Methods section.*
☒	☐	A description of all covariates tested
☒	☐	A description of any assumptions or corrections, such as tests of normality and adjustment for multiple comparisons
☐	☒	A full description of the statistical parameters including central tendency (e.g. means) or other basic estimates (e.g. regression coefficient) AND variation (e.g. standard deviation) or associated estimates of uncertainty (e.g. confidence intervals)
☒	☐	For null hypothesis testing, the test statistic (e.g. *F*, *t*, *r*) with confidence intervals, effect sizes, degrees of freedom and *P* value noted<br>*Give P values as exact values whenever suitable.*
☒	☐	For Bayesian analysis, information on the choice of priors and Markov chain Monte Carlo settings
☒	☐	For hierarchical and complex designs, identification of the appropriate level for tests and full reporting of outcomes
☒	☐	Estimates of effect sizes (e.g. Cohen's *d*, Pearson's *r*), indicating how they were calculated

Our web collection on statistics for biologists contains articles on many of the points above.

Software and code

Policy information about availability of computer code

Data collection	Topspin (Bruker Biospin) was used for collection of NMR data. GROMACS 2019.2 was used for generation of MD trajectories.
Data analysis	Initial NMR data analysis is performed with Topspin 4 (Bruker Biospin) and post-processing using INFOS (infos.sourceforge.net, provided by A. Smith) running in MATLAB (Mathworks). Detector analysis (NMR/MD) and frame analysis (MD) is performed in pyDIFRATE, available on GitHub (https://github.com/alsinmr/POPC_frames_archive, provided by A. Smith). pyDIFRATE runs in Python, and in addition to the standard libraries, uses numpy, scipy, pandas, MDAnalysis, matplotlib, and cv2. 3D plotting uses ChimeraX (UCSB).

For manuscripts utilizing custom algorithms or software that are central to the research but not yet described in published literature, software must be made available to editors and reviewers. We strongly encourage code deposition in a community repository (e.g. GitHub). See the Nature Portfolio guidelines for submitting code & software for further information.

Data

Policy information about availability of data

All manuscripts must include a data availability statement. This statement should provide the following information, where applicable:

- Accession codes, unique identifiers, or web links for publicly available datasets
- A description of any restrictions on data availability
- For clinical datasets or third party data, please ensure that the statement adheres to our policy

NMR relaxation data generated in this study is tabulated in the Supplementary Information and in the Source Data file. Processed data found in main text and supplementary figures can be found in the Source Data file. MD trajectories generated in this study have been deposited in the Zenodo database with 10 ns resolution (https://www.doi.org/10.5281/zenodo.564503). MD trajectories stored at 5 ps resolution are available upon request to the authors, where restricted

access is due to the large size (several terabytes) of the trajectories. Python code and partially processed MD data is provided on GitHub to assist in reproducing the analyses demonstrated in this study (https://www.doi.org/10.5281/zenodo.5642560)

Field-specific reporting

Please select the one below that is the best fit for your research. If you are not sure, read the appropriate sections before making your selection.

☒ Life sciences ☐ Behavioural & social sciences ☐ Ecological, evolutionary & environmental sciences

For a reference copy of the document with all sections, see nature.com/documents/nr-reporting-summary-flat.pdf

Life sciences study design

All studies must disclose on these points even when the disclosure is negative.

Sample size	Not applicable. In NMR, electronic noise is the main source of statistical variation, controlled for by using repeated scans for each experiment. Number of scans/experimental repetitions may be found in the Methods section. In MD, undersampling of the conformational space leads to statistical variation, controlled for by simply collecting very long trajectories (8.4 µs for our primary data set, yielding 1.68 million frames).
Data exclusions	The first 500 ns of each MD simulation were excluded from analysis to allow equilibration of the system
Replication	NMR experiments were each measured 2-4 times. Fitting multiple experiments also confirms reproducibility, because the measured parameters themselves are not independent among the different experiments. MD simulations were run with different number of POPC molecules (256, 1024, 4096).
Randomization	Random group sampling is not relevant to an NMR/MD study of a system's dynamics.
Blinding	Blinding is not relevant to an NMR/MD study of a system's dynamics.

Reporting for specific materials, systems and methods

We require information from authors about some types of materials, experimental systems and methods used in many studies. Here, indicate whether each material, system or method listed is relevant to your study. If you are not sure if a list item applies to your research, read the appropriate section before selecting a response.

Materials & experimental systems

n/a	Involved in the study	
☒	☐	Antibodies
☒	☐	Eukaryotic cell lines
☒	☐	Palaeontology and archaeology
☒	☐	Animals and other organisms
☒	☐	Human research participants
☒	☐	Clinical data
☒	☐	Dual use research of concern

Methods

n/a	Involved in the study	
☒	☐	ChIP-seq
☒	☐	Flow cytometry
☒	☐	MRI-based neuroimaging
